# Computational Systems Analysis of Dopamine Metabolism

**DOI:** 10.1371/journal.pone.0002444

**Published:** 2008-06-18

**Authors:** Zhen Qi, Gary W. Miller, Eberhard O. Voit

**Affiliations:** 1 Department of Biomedical Engineering, Georgia Institute of Technology and Emory University Medical School, Atlanta, Georgia, United States of America; 2 Department of Environmental and Occupational Health, Rollins School of Public Health, Emory University, Atlanta, Georgia, United States of America; 3 Center for Neurodegenerative Disease, Emory University School of Medicine, Atlanta, Georgia, United States of America; Chiba University Center for Forensic Mental Health, Japan

## Abstract

A prominent feature of Parkinson's disease (PD) is the loss of dopamine in the striatum, and many therapeutic interventions for the disease are aimed at restoring dopamine signaling. Dopamine signaling includes the synthesis, storage, release, and recycling of dopamine in the presynaptic terminal and activation of pre- and post-synaptic receptors and various downstream signaling cascades. As an aid that might facilitate our understanding of dopamine dynamics in the pathogenesis and treatment in PD, we have begun to merge currently available information and expert knowledge regarding presynaptic dopamine homeostasis into a computational model, following the guidelines of biochemical systems theory. After subjecting our model to mathematical diagnosis and analysis, we made direct comparisons between model predictions and experimental observations and found that the model exhibited a high degree of predictive capacity with respect to genetic and pharmacological changes in gene expression or function. Our results suggest potential approaches to restoring the dopamine imbalance and the associated generation of oxidative stress. While the proposed model of dopamine metabolism is preliminary, future extensions and refinements may eventually serve as an *in silico* platform for prescreening potential therapeutics, identifying immediate side effects, screening for biomarkers, and assessing the impact of risk factors of the disease.

## Introduction

Parkinson's disease (PD) is the most common neurodegenerative movement disorder, affecting more than 1% of the worldwide population over the age of 65 [1], [2]. Pathologically, PD is characterized by a progressive loss of dopamine neurons in the *substantia nigra pars compacta*, the presence of ubiquitin- and alpha-synuclein-positive cytoplasmic inclusions known as Lewy bodies [3], [4], depigmentation of the locus ceruleus, and autonomic dysfunction including sympathetic denervation of the heart [5]. While PD is a complex, multi-faceted disease, it has been suggested that neurodegeneration is primarily due to the generation of toxic species and to oxidative stress caused by abnormal dopamine metabolism [6]–[9]. Because loss of dopamine is responsible for the majority of the motor symptoms of PD, treatment options have mostly been based upon restoration of dopamine function by replacement of dopamine precursors, inhibition of degradative enzymes, or dopamine agonists. Some efforts have also been targeted toward the development of drugs for PD based on the synergistic action of dopamine, glutamate, and acetylcholine neurotransmission on GABAergic neurons in the striatum [10]–[14].

For many years, there has been considerable debate as to whether L-DOPA, administered to treat the symptoms of PD, may actually be exacerbating the disease due to oxidation of L-DOPA and its metabolites. In addition, L-DOPA treatment, which should counteract decreases in dopamine, tends to become ineffective after a while, again demonstrating the complexity of the controlled, adaptive metabolic system. Given the inherent complexity of dopamine dynamics and the redox state of the neuron, a quantitative analysis using mathematical models could enhance our understanding of these complicated processes.

To our knowledge, no dynamic model of presynaptic dopamine homeostasis is available outside the one proposed here. Some investigators have developed models for various aspects of dopamine function [15], [16], while others have elucidated some of the context in which dopamine metabolism affects PD, schizophrenia, opium addiction and other pathologies with focus on functions and processes on postsynaptic site, and specifically the role of DARPP-32 [14], [17]–[20]. We therefore set out to design a mathematical model of dopamine metabolism/homeostasis *de novo*. The focus on dopamine was chosen not only because of its common role in Parkinson's disease and other disorders, but also because of its key role as a physiologically, pathologically, and pharmacologically interesting neurotransmitter. The resulting model turned out to provide qualitatively reasonable and even semi-quantitatively accurate predictions of critical systemic responses of the dopamine metabolism and may eventually serve as a computational platform for rational drug development and biomarker screening for PD.

## Materials and Methods

The design of a pathway model requires three sets of input information: knowledge or assumptions regarding the pathway topology and regulation; choice of a suitable mathematical modeling framework; and data permitting parameter estimation.

### Pathway Structure

**Figure 1:**
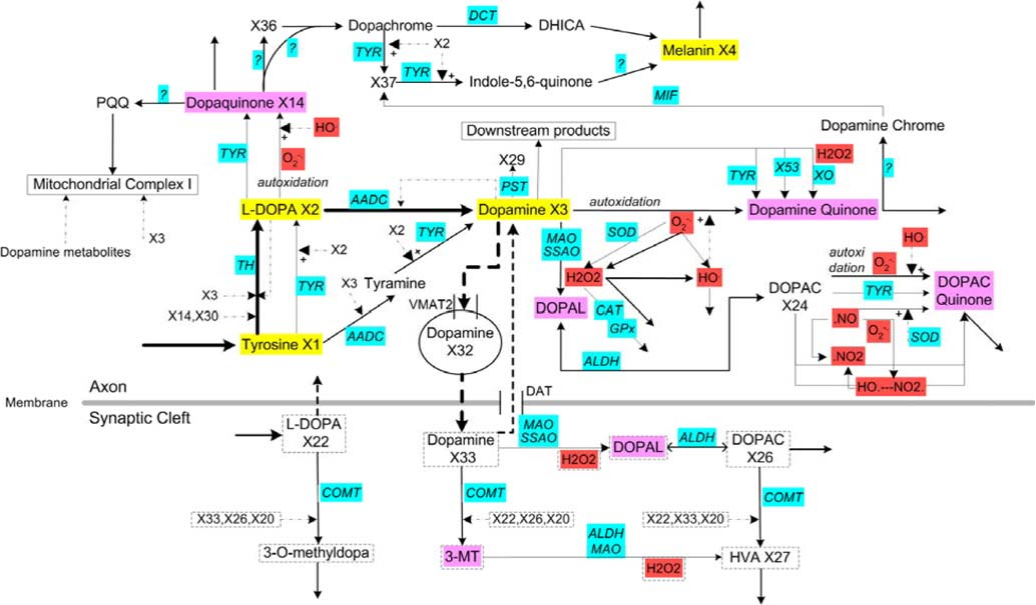
Simplified diagram of the nigrostriatal dopamine pathway, constructed from information in the literature, databases, and expert opinion of biochemists and neurologists. Detailed lists of all metabolites, variable names in the model, abbreviations, and numerical values are presented in Supplement Tables S1 and S2. Primary metabolites are highlighted in yellow, reactive oxygen and nitrogen species in light red, and toxic species in light purple; *X*_29_ is dopamine-3-sulfate, which is merely a recipient of material and not explicitly modeled; it is therefore not listed in Supplement Table S1 and S2. The ellipse shows dopamine inside vesicles. Metabolites in the synaptic cleft are indicated by dashed frames. Solid arrows represent biochemical reactions; associated enzymes are denoted in capital italics in light blue. Dash-dotted arrows designate inhibition, while dashed arrows with plus sign designate activation. Transport steps are represented as dashed arrows. Abbreviations of enzymes are: *TH* - tyrosine hydroxylase, *TYR* - tyrosinase, *XO* - xanthine oxidase, *ALDH* - aldehyde dehydrogenase, *MAO* - monoamine oxidase, *SSAO* - semicarbazide-sensitive amine oxidase, *AADC* - DOPA decarboxylase, *DCT* - dopachrome isomerase, *CAT* - catalase, *SOD* - superoxide dismutase, *COMT* - catechol O-methyltransferase, *GPx* - glutathione peroxidase, *MIF* - migration inhibitory factor. Question marks refer to enzymes that are unknown or not fully understood. Not shown in the diagram are deacetylipecoside, deacetylisoipecoside, noradrenaline, norcoclaurine, and norlaudanosoline; they are identified as “downstream products”.

As a starting point, we focused on the nigrostriatal pathway, which is the dopamine pathway most affected in PD. The simplified pathway diagram (Figure 1) was constructed by integrating information from databases [21], [22], literature [23], [24], and expertise provided by neurologists and biologists. Dopamine metabolism is located primarily in the presynaptic neuron and the synaptic cleft. Its homeostasis is controlled through a complicated biochemical network. Tyrosine, as the precursor of the dopamine pathway, is converted to L-DOPA by tyrosine hydroxylase (TH), which is regarded the rate-limiting enzyme of dopamine metabolism. DOPA decarboxylase (AADC) uses most of the L-DOPA to synthesize the key neurotransmitter dopamine, but L-DOPA can also be converted into the neuronal pigment melanin. Dopamine is packed into vesicles by the vesicular monoamine transporter (VMAT2). The packed dopamine is subsequently released into the synaptic cleft, where released dopamine can bind to dopamine receptors located on the postsynaptic membrane. Alternatively, dopamine can be taken up by the dopamine transporter (DAT) and returned back to the cytoplasm of the presynaptic neuron. Furthermore, extracellular dopamine can be methylated by catechol O-methyltransferase (COMT) to 3-methoxytyramine (3-MT). Monoamine oxidase (MAO) can oxidize cytoplasmic dopamine to 3,4-dihydroxyphenylacetate (DOPAC), which COMT may convert to homovanillate (HVA) .

### Modeling Framework

For our modeling environment we chose Biochemical Systems Theory (BST) [25]–[27], because it permits mathematical analyses and simulations of biochemical pathways under a minimal set of assumptions and even if crucial quantitative information is lacking, as it has been demonstrated in other, similarly complex contexts [28]–[30]. BST has been described numerous times, and detailed reviews are available [28]–[33]. The easy access to documentation of theory and applications allows us to minimize the description here; some pertinent details are given in the Supplemental Materials S1.

The key feature of BST is the representation of processes with products of power-law functions. This particular formulation is solidly anchored in Taylor's approximation theory and exhibits three important features. First, the representation is guaranteed to be appropriate in the vicinity of some chosen nominal point at which the system normally operates. Second, experience has shown that this vicinity can be quite large in biological systems and that power-law representations are often sufficiently accurate for high-percent or even fold variations in system components. In other words, systems characterized by high variability are often well characterized by power laws. Third, the resulting equations are very rich in structure and can model, in principle, any conceivable nonlinearity that has continuous derivatives [34], including limit cycles and deterministic chaos [35].

It is customary in BST to distinguish dependent variables (*X_i_*, *i* = 1, 2, …, *n*), representing genes, proteins, metabolites, or other components characterizing the dynamics of the system, from independent variables (*X_i_*, *i* = *n*+1, *n*+2, …, *n*+*m*), such as constant inputs or enzyme activities, that do not change during any single experiment. Both types of variables enter the appropriate power-law terms of the system, but equations are only formulated for the dependent variables. In the so-called Generalized Mass Action (GMA) form, which we use here, a BST model thus has the format
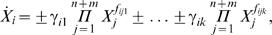
(1)where each power-law term is composed of a rate constant γ and of all variables that directly affect the modeled process, raised to a *kinetic order* exponent *f*. A rate constant characterizes the flux rate between pools or variables, while a kinetic order reflects the strength of the effect that the corresponding variable *X_j_* has on a given process.

If the true functions for the processes in the system are unknown, the numerical values of the parameters in the power-law representations (Eq. 1 and Supplement Eq. S1) are not known. Nevertheless, the structure of the equations is completely predictable and can be formulated symbolically from information about which variables directly affect each process. This type of information is often, though not always, available for metabolic pathways, and the task of determining appropriate parameter values remains to be one of the most significant challenges of modeling with BST or any other model.

### Parameter Estimation

Any numerical implementations and simulations of a BST model require the identification of parameter values. Although numerous methods have been developed over the years [30], [36], each new pathway creates its own challenges. To some degree, kinetic information may be available in enzyme databases [21], [22], but it is still often difficult to assess to what degree kinetic information from one organism and one set of (typically *in vitro*) conditions is applicable to another organism and possibly *in vivo*.

Our task of developing a numerical model of dopamine metabolism in the human brain (Fig. 1) did not allow us to use much published data. For instance, very little information is available on the exact concentrations of the metabolites that contribute to dopamine metabolism. Fortunately, every parameter in a BST model has a unique and unambiguously defined role, which greatly facilitates model design and estimation. This is to be seen in contrast to traditional kinetic models, which may contain multiple parameters characterizing the same process or event. For instance, detailed models of enzyme catalyzed mechanisms, such as a ping-pong mechanism, may require dozens of affinity, equilibrium, and rate constants that are associated with intermediate complexes, as poignantly discussed in Schultz [37]. Adding to this complication is the fact that it is seldom *a priori* clear which traditional mechanism would be most appropriate in a given situation. In BST models, by contrast, the effect of any given system component on any given process is uniquely described by one kinetic order plus one rate constant for the overall turn-over rate of the process. These differences between traditional and BST models are crucial for the estimation of parameters, because: (1) it is immediately clear how many parameters are to be used and how they enter the system of equations; (2) typically fewer parameters are to be estimated; and (3) the specific meaning of each parameter allows the setting of biologically supported constraints. In addition, experience with BST and other approaches suggests that systems models are rather robust if the system structure is captured correctly. In other words, if all connections between metabolites and all signals are accounted for, the parameter values are not as critical as one may think, and if a kinetic order is set as 0.75 instead of 0.6 or 1, the model responses are often still meaningful. All these aspects render BST a powerful structure for model implementation and estimation in the face of uncertainty.

Even with the stated advantages of BST, parameter estimation difficult. In fact, it may well be the hardest step in the entire modeling process. In light of the generic difficulties and the relative robustness of BST models, we decided to construct our dopamine model as a “relative” model based on expert knowledge. Specifically, adapting strategies for assessing complex systems from the fields of toxicology, risk assessment and evidence-based medicine, we asked several experts on neurochemistry and Parkinson's disease about the relative amounts of compounds in the dopamine system with respect to dopamine itself or to some other, relatively well characterized compounds in the system. We utilized this expert knowledge to estimate the relative metabolite profile at steady state as well as the relative magnitudes of fluxes within the dopamine system. We complemented this information with default values for kinetic orders, as they have been used in BST for a long time (Chapter 5 in [30]). It is clear that this type of procedure is not as quantitative as we would like. However, there is really not much of an alternative, and the models thus constructed do reflect expert opinions of the dopamine metabolism quite well [38]–[50]. As far as we know, the type of expert-based parameter estimation applied here has not been used in metabolic modeling before. To limit the parameter space further, we assumed that all processes are of first order with respect to the catalyzing enzyme, which is the implicit default assumption in most kinetic models.

The results of our parameter search are reflected in Supplement Table S1 and in the numerical models (see Supplemental Material S2). The independent variables of the model are listed in Supplement Table S2.

## Results

### Steady-State Analysis

The model of dopamine metabolism (represented in diagram form in Figure 1) was diagnosed, analyzed, and refined according to the guidelines provided in BST [30]. Due to the expert-based determination of parameter values, the steady-state concentrations and fluxes of the model were automatically consistent with expert opinion (Supplement Table S1). Also consistent with the expert-based flux profile, the rate constants associated with the generation of L-DOPA from tyrosine, conversion of L-DOPA to dopamine, and dopamine transportation turned out to have the largest magnitudes.

The dopamine model is locally stable and thus able to withstand small perturbations. Upon perturbations to the system, some metabolites may exhibit well-damped, small-amplitude oscillations. Such oscillations occur primarily in dopamine quinone (DA-Q), dopamine chrome, 5,6-dihydroxyindole-2-carboxylate (DHICA), melanin, DOPAC, and DOPAC quinone (DOPAC-Q).

### Simulation Analysis

The real testing of the model occurred through simulations of enzyme manipulations. In these simulations, local perturbations (on enzyme activities and regulatory functions) were introduced and the global model responses were compared to experimental findings. In contrast to the confirmation of steady-state features, which were used for model design, the consistency in these dynamic experiments is by no means automatic, and because there was no additional data fitting, the results are much more indicative of the quality of the model or of its shortcomings.

TH, COMT, DAT, and VMAT2 were selected as primary targets of manipulations, because experimental data are available for comparison. Simulations addressed heterozygotes (single allele deletion), gene knockouts (two allele deletion), gene hypomorphs (severely impaired transcription), as well as DAT inhibition. Striatal levels of dopamine and its two main metabolites, DOPAC and HVA, were compiled and used for comparison with model predictions.

Table 1 shows comparisons of experimental data and model predictions. Here, the activities of TH and COMT were changed through the use of heterozygote or homozygote knockouts, and cases with or without DAT inhibition [51]–[53] were tested against the resultant changes in the concentrations of dopamine, DOPAC, and HVA. The manipulation of VMAT2 included an approximate 95% genetic reduction of expression [38]. The results demonstrate a surprisingly high degree of accuracy of prediction, which supports the qualitative validity of our model. For example, relative changes in dopamine, DOPAC, and HVA levels in response to a COMT heterozygote mutation together with 90% DAT inhibition were predicted by the model as 15%, 90%, and −43%, respectively, while experimental measurements yielded comparable values of 22%, 71%, and −17%. Recent findings show that reduction of VMAT2 causes a severe reduction of dopamine, nigrostriatal neurodegeneration, increased vulnerability to various toxicants, and motor behavior deficits [50], [54], [55]. Our experimental data and model predictions revealed that reduced VMAT2 causes adverse effects such as lowering dopamine level, elevating toxic metabolites (cysteinyl adducts, a marker of quinone formation as seen in reference [38]), and exacerbating oxidative stress [38]. Equally supportive results were found for most of the other manipulations, where the model provided qualitatively correct and even semi-quantitatively accurate predictions of systemic behaviors.

**Table 1:** Changes in metabolite concentrations, relative to wild type, in response to manipulations of components of dopamine metabolism.

Manipulation	Metabolites	Experimental Result	Prediction
**TH heterozygote**	dopamine	No change	−2.68%
	DOPAC	No change	0.78%
	HVA	No change	−0.94%
**TH knockout**	dopamine	−99.58%	−100.00%
	DOPAC	Not detected	−100.00%
	HVA	Not detected	−100.00%
**COMT heterozygote**	dopamine	6.93%	18.56%
	DOPAC	10.54%	18.93%
	HVA	−14.52%	−49.10%
**COMT knockout**	dopamine	10.64%	37.39%
	DOPAC	232.95%	464.06%
	HVA	−100.00%	−100.00%
**COMT heterozygote+90% DAT inhibition**	dopamine	21.97%	14.57%
	DOPAC	71.46%	89.78%
	HVA	−17.01%	−43.36%
**COMT knockout+90% DAT inhibition**	dopamine	30.58%	30.25%
	DOPAC	447.50%	876.77%
	HVA	−100.00%	−100.00%
**VMAT2 LO** #	dopamine	−85.42%	−89.98%
	DOPAC	−58.00%	−28.96%
	HVA	−58.17%	−83.55%

# : VMAT2 LO mice show 95% reduction in the VMAT2 level compared to wild type mice.

Some predictions, especially with respect to HVA, differ considerably from experimental data. This relatively low accuracy is in most cases due to our deliberate decision not to tweak parameter values arbitrarily. Specifically, all enzymes have kinetic orders of 1, reflecting the typical default of first-order involvement. Deviations from this default could effectively improve accuracy of prediction of HVA level. For example, if one simply alters the kinetic order of COMT in the synthesis of HVA to 0.3, the relative change on HVA in the COMT heterozygote and COMT heterozygote/DAT 90% inhibition experiments is predicted to be −20% and −17%, which is very similar to the experimental findings (−15% and −17%). It is unknown at this point whether there is rationale for such an alteration, but the change in kinetic order indicates that the model could be slightly reparameterized for more accurate numerical results. We did not consider this necessary at the present state of our model.

### Gain Analysis

An important feature in any systems analysis is the relative strength of control that each part of the system plays. Questions of this type are addressed with methods of sensitivity analysis, where one investigates output responses of the system due to small, permanent disturbances, either in the environment or in the system structure, which in turn is characterized by its parameters. These kinds of sensitivity analyses allow a preliminary screening for environmental factors and system properties that are most critical or that could potentially be manipulated efficaciously in order to alter important system behaviors, such as disease states. Interpreted correctly, sensitivity analyses can aid both in the discovery of biomarkers and the development of potential pharmacological interventions.

It is customary in BST to distinguish the model's steady-state sensitivity with respect to parameter values as opposed to independent variables. The relative change of a dependent variable at steady state in response to a relative change in an independent variable is specifically called a logarithmic gain (log gain). Each gain or sensitivity value corresponds to the (positive or negative) percent change evoked by a 1% increase in an independent variable or a parameter.

Log gains are very useful for the assessment of the robustness of a system, because changes in independent variables often reflect natural fluctuations within the internal or external milieu of the organism. In our model, independent variables include dozens of enzymes and environmental variables that are considered constant and at their normal value under physiological conditions (Supplement Table S2). Among these, L-Glutamate (Glu) can be viewed as a toxic species that is potentially deleterious to neurons. ^.^NO is a reactive nitrogen species (RNS), while glutathione (GSH) and ascorbate (ASB) are antioxidants that can alleviate oxidative stress. The concentrations of these variables depend on diet and the environment and are thus subject to repeated dynamic changes. Other independent variables mainly represent enzyme activities that are not directly linked to environmental or dietary fluctuations, but are immediately affected by genetic variations and possibly during disease.

**Figure 2:**
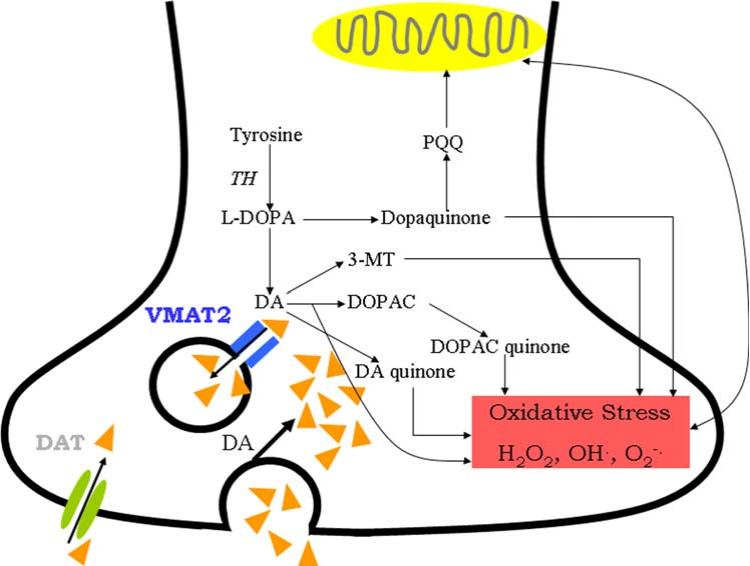
Interrelationships between dopamine metabolism, VMAT2, DAT, the generation of toxic species, oxidative stress and mitochondrial dysfunction in Parkinson's disease.

Log gains were analyzed for all combinations of dependent and independent variables and generally found unremarkable. The entire set of gains is large, and Supplement Table S3 only shows the most pertinent results. Of primary interest are gains of important metabolites, reactive oxygen species (ROS), RNS, and toxic species that are closely associated with the pathogenesis of PD (Fig. 2). Metabolites of special interest include L-DOPA, dopamine, dopamine in vesicles (DA-v), extracellular dopamine (DA-e), DOPAC, extracellular DOPAC (DOPAC-e), HVA, and melanin. ROS include O_2_^−.^, H_2_O_2_, extracellular H_2_O_2_ (H_2_O_2_-e), and HO^.^, while RNS comprise HO^.^—NO_2_^.^ and ^.^NO_2_. Toxic species in our model are dopaquinone (DOPA-Q), 3-MT, DOPAL, extracellular DOPAL (DOPAL-e), DOPAC-Q, and DA-Q.

As Supplement Table S3 indicates, all gains are relatively small in magnitude, which is typically considered a positive sign of model robustness, but also implies that it is difficult to change concentrations (such as for dopamine) through induced alterations in enzyme activities. Most of the gains are small in magnitude (<<1) so that fluctuations are effectively attenuated in almost all cases. Among the dozens of independent variables, only AADC has a noticeable effect on DOPA concentration, with a log gain of −1.63%, which is to be interpreted as a 1.63% relative decrease in response to a 1% increase in AADC activity. Dopamine in all locations (intracellular, in vesicles, or extracellular) is negatively affected by increases in the activities of MAO and semicarbazide-sensitive amine oxidase (SSAO), which are enzymes catalyzing the degradation of dopamine. Outside MAO and SSAO, the vesicular monoamine transporter (VMAT2) also affects the concentration of DA-v and DA-e, but with positive log gains; DAT and COMT have a slightly negative effect on DA-e. With respect to DOPAC, only Fe^2+^ has a relatively significant influence with a log gain of −0.74%. Increases in many independent variables, such as S-Adenosyl-L-methionine (SAM), DAT, MAO, SSAO, and COMT, negatively affect DOPAC-e. DOPAC-e is positively affected by increases in VMAT2, extracellular monoamine oxidase (MAO-e), and extracellular semicarbazide-sensitive amine oxidase (SSAO-e). HVA is mainly influenced by SAM and COMT. Melanin, the source of pigmentation in dopaminergic neurons, can be altered by changing the concentration of MAO, SSAO, or COMT.

Oxidative stress may be assessed approximately by measuring concentrations of ROS and RNS, and its association with dopamine metabolism is reflected in the corresponding log gains (Supplement Table S4). All are close to 1 or smaller in magnitude, identifying them as unremarkable and difficult to affect.

One might ask to what degree it could be possible to reduce the amounts of toxic species, such as DOPA-Q. Supplement Table S5, exhibiting log gains with respect to these metabolites, indicates that toxic species are difficult to remove. The relatively most effective way of decreasing DOPA-Q would be an increase in the activity of AADC with a log gain of 3.37. Reductions in Fe^2+^ or increase of COMT activity would have a similar but weaker effect. Unduly high values of 3-MT could possibly be alleviated by activation of extracellular aldehyde dehydrogenase (ALDH-e) or MAO-e, or through inhibition of SAM or COMT. Increases in VMAT2, SAM, MAO, SSAO, or COMT could moderately lessen content of DA-Q. According to the gain analysis, it would be very difficult to reduce the concentration of DOPAC-Q, because all gains are close to zero.

While all log gains are small, it is still worth exploring the effects of combined and larger alterations in some of the variables with relatively higher gains. Most metabolites of the dopamine pathway have low concentrations in the human brain, so that even small amounts of medication would evoke relatively large deviations and could thereby be quite efficacious. Some results of such an exploration are shown in Table 2.

**Table 2:** Alterations in metabolite concentrations, relative to wild type, in response to MAO inhibition and increase of VMAT2.

Metabolites	50% MAO Inhibition	50% VMAT2 Increase	50% MAO Inhibition+50% VMAT2 Increase	10% MAO Inhibition+50% VMAT2 Increase	10% MAO Inhibition+100% VMAT2 Increase
**DA-i**	131.30%	−8.72%	116.47%	5.29%	−2.15%
**DA-e**	160.98%	43.99%	275.86%	69.83%	115.41%
**DA** #	131.51%	36.38%	223.69%	57.34%	94.59%
**H_2_O_2_**	−30.64%	−8.92%	−36.22%	−12.85%	−19.33%
**H_2_O_2_-e**	26.61%	17.95%	44.04%	21.64%	36.45%
**HO^.^**	−52.51%	−17.48%	−59.98%	−24.57%	−35.60%
**^.^NO_2_**	−15.17%	−9.25%	−21.99%	−10.98%	−17.51%
**DOPA-Q**	145.25%	−27.94%	87.68%	−13.49%	−33.52%
**3-MT**	732.53%	−5.49%	719.03%	31.73%	27.26%
**DOPAC-Q**	−42.17%	−8.72%	−45.88%	−14.72%	−20.74%
**DA-Q**	142.15%	−27.35%	85.97%	−15.16%	−34.87%

# : Total amount of dopamine (intracellular, intravesicular, and extracellular).

Table 2 shows that increasing VMAT2 could elevate concentrations of extracellular dopamine and total dopamine, but is less efficacious than MAO inhibition. However, MAO inhibition has the undesired side effect of elevating some of the toxic species, such as 3-MT, DA-Q, and DOPA-Q. Severe increases in these toxic species are deleterious to dopaminergic neurons and may induce neuronal degeneration. The combined targeting of VMAT2 and MAO shows a substantial increase of dopamine while keeping the concentrations of toxic species under control. For example, 10% MAO inhibition together with a 50% increase in VMAT2 is predicted to elevate extracellular dopamine by 70%, while elevating 3-MT by only 32%. The latter has to be seen in contrast to 50% MAO inhibition alone, which raises 3-MT more than 7-fold. The other toxic species are actually lowered by the combination regimen. While these results are based on a preliminary model, they indicate how a computational systems approach may aid the screening and selection of pharmacological therapies.

### Parameter Sensitivity Analysis

The parameter set of the dopamine metabolic model is comprised of kinetic orders and rate constants. Each kinetic order is a reflection of the strength with which a variable affects the corresponding process, while a rate constant determines the turnover rate of a process [25], [26]. As in the case of log gains, a sensitivity value of (positive or negative) *p* indicates a *p*% change in some outcome measure due to a 1% increase in the parameter of interest.

Given the large number of dependent variables and parameters, the full set of sensitivities is immense and rather uninteresting. Indeed, most sensitivity values are small, indicating that moderate perturbations in model structure are essentially inconsequential. The highest sensitivities (in a range of magnitude 5 to 10) are found with respect to DOPAC-e. A pertinent selection of somewhat large sensitivities is presented in Supplement Table S6. As an example, the kinetic orders associated with dopamine degradation show negative gains for dopamine [−3.8 to −5.4], as well as for DOPA [−2.1 to −2.9], DA-v [−3.8 to −5.4], DA-e [−4.5 to −6.4], DOPAC-e [−7.3 to −10.2], and melanin [−3.8 to −5.3]. As another example, the sensitivity profile of DA-e ranges from 0 to about 6.4 in absolute magnitude. DA-e is the actual transmitter of nerve signals for movement control. Its only noteworthy sensitivities (with respect to DA-e self-degradation and dopamine degradation toward DOPAL) are negative. Hence, measures to decrease the effects of dopamine, MAO, or SSAO on the reaction between dopamine and DOPAL could potentially constitute efficacious interventions to increase DA-e concentrations. DOPAC-e is negatively influenced by dopamine degradation and positively by increases in the reaction between DA-e and DOPAL-e.

As with kinetic orders, most of the sensitivities with respect to rate constants are negligible (Supplement Table S7). An exception is rate constant γ_1_0_, which represents the exogenous input flux into the dopamine metabolic system. As one might expect, enhancements in this flux yield increases in the concentrations of most primary metabolites especially that of melanin, with the exception of DOPAC, which slightly decreases. Almost all other rate constant sensitivities are of magnitude 1 or smaller.

The kinetic order and rate constant sensitivities of ROS and RNS are shown in Supplement Tables S8 and S9. As with enzymes and antioxidants within the cell defense system, such as CAT, SOD, GPx, and GSH, their kinetic order sensitivities show moderate or higher capability of scavenging those ROS and RNS. As before, the influx to the system generally has an enhancing effect. Not surprisingly, the sensitivities suggest that increased degradation of dopamine would increase all ROS and RNS except for H_2_O_2_-e, while most of ROS and RNS could be moderately alleviated by enhancing their own degradations.

Parameter sensitivities with respect to toxic species are presented in Supplemental Tables S10 and S11. Changes in quite a few kinetic orders could yield decreases in the concentration of DOPA-Q, especially those parameters associated with L-DOPA conversion to dopamine and DOPA-Q degradation to pyrrolo-quinoline quinine (PQQ). 3-MT, DOPAL, and DOPAC-Q are most strongly affected by changes in kinetic orders associated with their own degradations. Increases in kinetic orders for the conversion of dopamine to DOPAL could result in a substantial reduction of toxic DA-Q.

Changes in the rate constant γ_1_0_ of the input flux have concomitant effects on DOPA-Q, DOPAL-e, and DA-Q and increasing the rate of the degradation of toxic species would moderately alleviate their concentrations. Other than that, the system is by and large buffered against changes in turnover rates.

## Discussion

The ability of dopamine replacement to restore the primary movement deficit in PD is striking. Unfortunately, the success of this treatment is temporary with side effects limiting the effectiveness. An improved understanding of the dynamics and control of dopamine metabolism is necessary for improving approaches to dopamine restoration therapy. The main challenge of this endeavor is the sophistication and complexity of the dopamine pathway, which is further confounded by the enormous difficulties in laboratory measurement *in vivo*. Our education and Western culture have trained us to subdivide complicated problems into manageable tasks, and this strategy is very successful if the system under investigation is linear. However, for systems containing many nonlinear processes, as it is the case with dopamine metabolism, dissection of the integrated system becomes problematic, because our brain is not able to weigh the relative importance of parallel or counteracting processes against each other in a quantitative fashion, or to assess the strength of a control signal against the magnitude of the flux through a pathway.

Drawing from the fields of neuroscience, neurology, and biochemistry, we collected a large body of information characterizing the connectivity and regulation of the dopamine pathway and converted this information into a BST model. As described before, we then numerically configured this model in accordance with expert knowledge on neurochemistry of the dopamine metabolism. Even though this procedure was at best semi-quantitative, it allowed us to set up a BST model that was consistent with expert knowledge. As a first step of model diagnosis, we analyzed systemic properties at the steady state. By design, the steady-state concentrations and fluxes reflected the input suggested by the experts. Not as automatic, but also not very surprising, the model turned out to be locally stable, and while some variables exhibited oscillations, these were of small amplitude and strongly damped. Much more interestingly, simulations of scenarios not used for model design and implementation turned out to be surprisingly close to experimental and clinical observations. Some examples are revisited below.

Our model results showing that the dopamine concentration in the brain increases if the enzyme MAO is inhibited are consistent with clinical observations. Selegiline, a MAO inhibitor, has long been used as a therapeutic treatment for PD [56]–[59]. Beyond the prediction on ultimate effects of MAO inhibition, our results also suggest that inhibition of MAO may have undesired side effects. In addition to augmenting dopamine levels, reduction of MAO activity increases the amounts of toxic species, which in turn may contribute to the degeneration of dopaminergic neurons. It is tempting to speculate that the less than expected beneficial effects of Selegiline seen in the DATATOP study [60] were due to these unforeseen deleterious effects of MAO inhibition.

Our results suggest that a combination of targets could be considered in the development of improved drugs for PD. The predicted adverse effects of lowering VMAT2 suggest that increases in VMAT2 or decreases of DAT in combination with MAO inhibition seem to have the potential of efficiently increasing extracellular dopamine levels while minimizing side effects caused by elevated toxic species such as 3-MT, DA-Q, and DOPA-Q induced by sole MAO inhibition (see Table 2). Such an example is just a small indication of the capability of a mathematical model in exploring potential pharmacological interventions.

According to our results, a reduction in VMAT2 activity causes DA-e and DA-v to decline. Meanwhile, the toxic species DOPA-Q and DA-Q are elevated. These findings were confirmed by a recent paper from our laboratory that showed mice with low VMAT2 expression display increased formation of toxic dopamine metabolites, increased oxidative damage in dopamine-rich areas, and a Parkinson's disease-like phenotype [54], again supporting the idea that increased VMAT2 could be beneficial in the treatment of Parkinson's disease. Indeed, a recent human study showed that genetic polymorphisms associated with increased VMAT2 expression reduced the incidence of PD in women [47]. According to the model, down-regulation of VMAT2, as well as up-regulation of DAT, can elevate the amount of melanin. This prediction is consistent with studies of Matsunaga and others showing that neuromelanin content is inversely correlated with neurological degeneration in PD patients [61], [62]. Neuromelanin has the ability to bind a variety of metal ions, especially iron. Elevation of iron, in turn, may lead to increasing concentrations of ROS and RNS, as predicted by our model, and especially to an elevated concentration of highly reactive hydroxyl radicals. Our predictions on the adverse effects of iron are also supported by several recent studies [63], [64].

An immediate use for the model is the determination of “choke points.” These are features of the system that, if slightly “loosened,” permit a higher flux of material. Primary targets of such an analysis in our case are extracellular dopamine, which should be increased to counteract the motor symptoms of PD, and toxic species, which should be minimized, for instance by means of decreased production or increased degradation. The starting point for the identification of choke points is the analysis of sensitivities and log gains. Essentially all sensitivities and gains in our model are small in magnitude, which is usually viewed as a good sign, because it affirms robustness of the model. However, low sensitivities and gains also imply that it is difficult to alter the system through subtle manipulations, for instance, of some of the enzymes in the system. The model analysis identified several kinetic orders with relatively high sensitivities, but because these reflect structural features of enzymatic processes, they are unlikely to present accessible targets for drug treatment. Among the rate constants, the rate of influx to the system (into the tyrosine pool) stands out as most significant. It remains to be seen whether this influx can be affected efficaciously by dietary or pharmaceutical intervention. However, its deleterious side effects, similar to those from L-DOPA administration, must be taken into account.

The most direct targets for intervention are exogenously supplied substrates and enzyme activities, which could at least theoretically be altered through inhibition or other mechanisms. These targets are usually represented in the model through independent variables, so that log gains are more important indicators than parameter sensitivities. In the model, almost all gains are relatively low, which again confers a certain degree of robustness and implies that small changes are not particularly consequential. However, pharmaceutical or dietary treatments do not have to be in the low-percent range, especially for brain metabolites that are present in very small concentrations. Thus, it could be possible to effect beneficial changes, for instance by a combined inhibition of MAO and DAT, or inhibition of MAO together with activation of VMAT2 (see Table 2).

It is clear that the model presented here is very preliminary. In addition to the uncertainties during parameter estimation, the model is based on a number of simplifying assumptions. For instance, several metabolites are treated as independent variables, even though they are certainly regulated at a different organizational scale and must be expected to change over long time horizons. These variables include tetrahydrobiopterin, Glu, SAM, prostaglandin G2, ^.^NO, GSH, ASB, N-acetylcysteine, Fe^2+^, Fe^3+^, NADH, NAD+, NADPH, NADP+, VMAT2, DAT, and ATP. Furthermore, some metabolites are not taken into account, even though they might be of importance for the proper functioning of the nigrostriatal dopamine pathway. Finally, some processes and enzymes within dopamine metabolism may be missing or are not fully understood (as indicated by question marks in Fig. 1). Thus, the proposed model is to be considered a starting point for more detailed and refined estimations. It may also be used as a preliminary input module for modeling approaches elucidating phenomena further downstream, as they are, for instance, related to DARPP-32 signaling [14], [20].

Although the model is preliminary, it exhibits a number of good features, such as robustness and consistency with diverse experimental and clinical observations. Its main utilization at this point is as an exploratory tool for generating hypotheses that are to be screened and tested later in animal models. These hypotheses may refer to the discovery of environmental exposures, biochemical or genetic variations, and different disease trajectories toward PD. Because manipulations are infinitely easier in the model than in an animal experiment, numeric simulations and model analyses can be executed in a very short period of time, permitting comprehensive screening of possible scenarios and responses [65].

Our model was initially formulated for the nigrostriatal dopamine pathway and with emphasis on PD. However, with slight modifications it could be applicable to other disorders in which dopamine homeostasis is altered, as for instance in attention deficit hyperactivity disorder. The concepts and methodology in these cases will be unchanged, but adjustments are likely to be necessary with regard to the numerical properties of the model and possibly the topology of the underlying biochemical network.

## Supporting Information

Materials S1 Supplements without tables
(0.06 MB DOC)

Table S1 Metabolite concentrations and fluxes at steady state (relative units)
(0.06 MB DOC)

Table S2 List of independent variables (include environmental factors and enzymes)
(0.06 MB DOC)

Table S3 Among dozens of independent variables, only DOPA decarboxylase (AACD) significantly affects DOPA concentration, with a log gain of −1.63%, which means that DOPA is predicted to exhibit a 1.63% decrease in response to 1% increase in AADC activity. Dopamine, no matter where it is located (intracellular, in vesicles, or extracellular) is negatively affected by enhancement of MAO or semicarbazide-sensitive amine oxidase (SSAO), which are enzymes catalyzing the degradation of dopamine. Except for MAO and SSAO, the vesicular monoamine transporter (VMAT2) also influences the concentration of DA-v and DA-e, but with positive log gains, while DAT has a negative effect on DA-e. Only Fe2+ has a significant influence on DOPAC, with a log gain of −0.74%. Increases in many independent variables, such as S-adenosyl-L-methionine (SAM), DAT, MAO, SSAO, and COMT are predicted to affect DOPAC-e negatively, while VMAT2, extracellular monoamine oxidase (MAO-e), and extracellular semicarbazide-sensitive amine oxidase (SSAO-e), DOPAC-e have positive log gains. HVA is mainly affected by SAM and COMT. Melanin, the source of pigment in dopaminergic neurons, is affected by changes in the concentrations of SAM, Fe^2+^, VMAT2, DAT, MAO, SSAO, or COMT. ^#^ Gain values are given in percent change due to a 1% percent change in an independent variable. ^*^ Gain s with absolute values less than 0.5 are discarded.
(0.05 MB RTF)

Table S4 Log gains of ROS and RNS in response to alterations in independent variables^#*^. Only some ROS and RNS show significant log gains with respect to the up-regulation of independent variables. For instance, the gain of O_2_^−.^ indicates a 1.29% relative decrease upon 1% elevation of SOD. H_2_O_2_ and H_2_O_2_-e could not be effectively changed by alterations in any of the independent variables. HO^.^ increased 1.46%, 1.13%, and 1.13% in response to 1% up-regulation of Fe^2+^, MAO, and SSAO, respectively. Increasing ^.^NO or Fe^2+^ could promote formation of HO^.^—NO_2_^.^ and ^.^NO_2_, while increases in ASB, CAT, SOD, COMT, or GPx alleviate HO^.^—NO_2_^.^. ^#^ Gain values are given in percent change due to a 1% percent change in an independent variable. ^*^ Gains with absolute values less than 0.5 are discarded.
(0.04 MB DOC)

Table S5 Log gains of toxic species with respect to alterations in independent variables^#*^. The most effective way of decreasing toxic DOPA-Q is increasing the activity of AADC; lowering Fe^2+^ has a similar but lesser effect. 3-MT could be alleviated by elevation of extracellular aldehyde dehydrogenase (ALDH-e) or MAO-e, or reduction of SAM or COMT. DOPAL is mainly affected by Fe^2+^, NAD^+^, NADH, NADP^+^, NADPH, and ALDH. Elevation of DAT, MAO, or SSAO has the most significant negative effect on the concentration of DOPAL-e, while increases in VMAT2, MAO-e, or SSAO-e could promote generation of DOPAL-e. None of these primary metabolites could significantly reduce the concentration of DOPAC-Q. To lessen content of DA-Q, Fe^2+^ should be decreased or VMAT2, SAM, MAO, SSAO, or COMT increased. However, all effects are only moderate. ^#^ Gain values are given in percent change due to a 1% percent change in an independent variable. ^*^ Gains with absolute values less than 0.5 are discarded.
(0.05 MB DOC)

Table S6 Sensitivity of primary metabolites in response to alterations in kinetic orders^#*^. DOPA shows negative gains with respect to kinetic orders for its degradation processes, except for the inhibition from dopamine. The sensitivity of DOPA in response to alteration of dopamine inhibition is 3.24%, which means 1% up-regulation of dopamine inhibition is predicted to cause a 3.24% relative increase in DOPA. Dopamine and DA-v have very similar sensitivity profiles for kinetic orders, with positive values associated with DOPA degradation and negative values associated with degradative processes of dopamine and DA-e (especially for fluxes from dopamine to DOPAL and from DA-e to 3-MT). DA-e is the actual transmitter of nerve signals for movement control. Its only noteworthy sensitivities (with respect to DA-e self-degradation and dopamine degradation toward DOPAL) are negative. Hence, measures to decrease the effects of dopamine, MAO, or SSAO on the reaction between dopamine and DOPAL could potentially constitute efficacious interventions to increase DA-e concentrations. DOPAC is mainly affected by kinetic orders for H2O2 degradation. Sensitivities of DOPAC-e are negatively influenced by dopamine degradation and positively by increases in the reaction between DA-e and DOPAL-e. HVA and melanin show several significant sensitivity values, mostly associated with kinetic orders related to the degradation of DOPA, dopamine, and DA-e. ^#^ Sensitivity values are given in percent change due to a 1% percent change in a parameter ^*^ Sensitivities with absolute values less than 0.5 are discarded.
(0.08 MB DOC)

Table S7 Sensitivity of primary metabolites in response to alterations in rate constants^#*^. As with kinetic orders, most of the sensitivities with respect to rate constants are negligible in magnitude. Of note is rate constant γ_1−0_, which represents the exogenous input flux into the dopamine metabolic system. As one might expect, enhancements in this flux yield increases in the concentrations of most of the primary metabolites especially that of melanin, with the exception of DOPAC, which slightly decreases. Almost all other rate constant sensitivities are of magnitude 1 or smaller. DOPA is negatively affected by rate constants for degradation of DOPA and dopamine. Dopamine, DA-v, and DA-e have negative sensitivities with respect to the rate constant for the reaction between dopamine and DOPAL. Increasing the transport of dopamine into vesicles could increase the concentrations of DA-v and DA-e, while enhancing degradation of DA-v and DA-e is expected to lead to decreases in their concentrations, respectively. DOPAC, DOPAC-e and HVA are mainly affected by rate constants related to their synthesis and degradation. DOPAC-e also has significant sensitivities with respect to rate constants for dopamine reactions. Many rate constants influence melanin to some extent but much less than γ_1−0_. ^#^ Sensitivity values are given in percent change due to a 1% percent change in a parameter. ^*^ Sensitivities with absolute values less than 0.5 are discarded.
(0.06 MB DOC)

Table S8 Sensitivities of ROS and RNS in response to alterations of kinetic orders^#*^. Enhancing the degradation of dopamine increases all ROS and RNS except H_2_O_2_-e, while elevation of kinetic orders for effluxes out of DA-e reduce the concentrations of most ROS and RNS. O_2_^-.^, H_2_O_2_, and H_2_O_2_-e decrease upon up-regulation of their relevant degradation processes. Increasing kinetic orders for degradation of DOPAC, DOPAC-e, O_2_^-.^, H_2_O_2_, or HO^.^ alleviates HO^.^ concentration. HO^.^ —NO_2_^.^ and ^.^ NO_2_ could be decreased by enhancing the degradation of O_2_^-.^ or H_2_O_2_. Enzymes and antioxidants within the cell defense system, such as CAT, SOD, GPx, and GSH, exhibit kinetic order sensitivities that show moderate capability of scavenging these reactive species.
(0.05 MB DOC)

Table S9 Increasing rate constant γ_1−0_ would raise the concentrations of all ROS and RNS, especially those of HO^.^ and HO^.^—NO_2_^.^; the rate constants for degradation of dopamine to DOPAL have similar but lesser effects. Most of ROS and RNS could be significantly alleviated by enhancing their own degradation. HO^.^ could also be reduced through enhancement of H_2_O_2_ degradation. Increasing the rate constant for DOPAC autoxidation would elevate HO^.^—NO_2_^.^ and ^.^NO_2_, while rate constants for O_2_^-.^ and HO^.^ degradation have the opposite effect.
(0.04 MB DOC)

Table S10 Sensitivity of toxic species in response to alteration of kinetic orders^#*^. Quite a few kinetic orders could efficaciously decrease the concentration of DOPA-Q, especially those related to L-DOPA conversion to dopamine and DOPA-Q degradation to Pyrrolo-quinoline quinine (PQQ). 3-MT, DOPAL, and DOPAC-Q have significant sensitivities with respect to kinetic orders related to their degradation. Reduction in kinetic orders for DA-e degradation to 3-MT could also effectively alleviate the concentration of 3-MT. Increasing the effect of dopamine, MAO, or SSAO on dopamine degradation could greatly alleviate the concentration of DOPAL-e; decreasing the kinetic order for the reaction between DA-e and DOPAL-e has a similar effect. Increases in DOPAL-e degradation could somewhat reduce its own concentration. DA-Q shows significant sensitivities mostly with respect to kinetic orders for the conversion of dopamine to DOPAL. Elevation of the action of GSH on H_2_O_2_ could indirectly result in great reductions of toxic DA-Q. ^#^ Sensitivity values are given in percent change due to a 1% percent change in a parameter. ^*^ Sensitivities with absolute values less than 3.0 are discarded.
(0.05 MB DOC)

Table S11 Sensitivity of toxic species in response to alteration of rate constants^#*^. Rate constant γ_1−0_, which accounts for the exogenous input flux into the dopamine system has a strong positive effect on the concentrations of DOPA-Q, DOPAL-e, and DA-Q. Enhancing the degradation of DOPA could decrease DOPA-Q moderately compared with that from γ_1−0_. To effectively reduce the concentrations of 3-MT, DOPAL, and DOPAC-Q, their relevant rate constants for degradative processes should be raised. DOPAL-Q could be reduced by increasing the rate constant for conversion of dopamine to DOPAL or slowing down degradation of DA-e to DOPAL-e. The rate constant for DA-Q degradation has a negative effect on the concentration of DA-Q but with smaller magnitude in comparison with γ_1−0_. ^#^ Sensitivity values are given in percent change due to a 1% percent change in a parameter ^*^ Sensitivities with absolute values less than 0.5 are discarded.
(0.06 MB DOC)
