# Supplementary material for: Computational Systems Analysis of Dopamine Metabolism
Source: PLoS One. 2008 Jun 18;3(6):e2444. doi: 10.1371/journal.pone.0002444 (PMC2435046; doi:10.1371/journal.pone.0002444)
Supplement: Materials S1 — Supplements without tables (0.06 MB DOC) [file pone.0002444.s001.doc]

Supplements

**Computational Systems Analysis of Dopamine Metabolism**

Zhen Qi, Gary W. Miller, Eberhard O. Voit

###### **S1. Mathematical model of dopamine metabolism in the human brain**

The model is formulated within the guidelines of Biochemical Systems Theory [1-3] and implemented in the freeware PLAS [4]. Equations for the model of dopamine metabolism are formulated in Generalized Mass Action (GMA) form, but we use for some computations the alternative BST format of an S-system, where the primary focus is on metabolites rather than on fluxes. Specifically, each metabolite has at most one input (which is composed of all incoming fluxes) and one output (which is composed of all outgoing fluxes) and each of the two is collectively represented as a product of power-law functions. The result is thus

, (Eq. S1)

which is somewhat simpler in structure and permits some algebraic steady-state analyses that are not possible with GMA models [2]. Because it is easy to convert Equation 1 into the corresponding Equation S1, many BST analyses often use GMA and S-systems in parallel.

To anchor the model, we searched the literature and queried biochemical and clinical experts. We implemented the model using this information, along with default values for kinetic orders (1 for enzymes, usually in the range of 0.5 and 1 for positive effects and between 0 and -0.5 for inhibitory effects by metabolites; see Voit, 2000: Chapter 5) and adjusted the rate constants so that the steady state of the model matched experts’ opinion. The result is given below in PLAS format.

**S2. PLAS implementation of the model of dopamine metabolism**

X1' = 1000 -12.34409 (X1^0.5) (X4^-0.3) (X5^-0.3) (X7^-0.3) (X16^-0.3) X35 -0.0005656854 (X1^0.5) (X4^0.5) X36 -0.01009356 (X1^0.5) (X7^-0.3) X42

X2' = 12.34409 (X1^0.5) (X4^-0.3) (X5^-0.3) (X7^-0.3) (X16^-0.3) X35 -300.4164 (X2^0.5)

X3' = 0.01009356 (X1^0.5) (X7^-0.3) X42 -0.001825742 (X3^0.5) (X4^0.5) X36

X4' = 12.34409 (X1^0.5) (X4^-0.3) (X5^-0.3) (X7^-0.3) (X16^-0.3) X35 +0.0002828427 (X1^0.5) (X4^0.5) X36 +2.767677 (X5^0.5) (X18^0.5) -6.670782 (X4^0.5) (X7^-0.3) X42 -0.3478394 X4 (X30^0.5)

X5' = 0.3478394 X4 (X30^0.5) +0.0002828427 (X1^0.5) (X4^0.5) X36 -24.75485 (X5^0.5) -2.767677 (X5^0.5) (X18^0.5)

X6' = 18.2567 (X5^0.5) -9.128352 (X6^0.5)

X7' = 6.670782 (X4^0.5) (X7^-0.3) X42 +0.001825742 (X3^0.5) (X4^0.5) X36 +4.198429 X9 -2.998878 X7 -0.001785214 (X7^0.5) X40 X41 -0.03710922 X7 (X30^0.5) -0.0009917856 (X7^0.5) X36 -0.0009917856 (X7^0.5) X37 -0.00044354 (X7^0.5) (X28^0.5) X38 -0.2975357 (X7^0.5)

X8' = 2.998878 X7 -0.03894646 X8

X9' = 0.03894646 X8 -0.6772013 (X9^0.5) (X12^-0.3) (X14^-0.3) X48 -0.001049607 (X9^0.5) X47 X51 -4.198429 X9

X10' = 0.6772013 (X9^0.5) (X12^-0.3) (X14^-0.3) X48 -0.004693986 (X10^0.5) X46 X47

X11' = 0.001049607 (X9^0.5) X47 X51 -2.642754 (X11^0.5) (X12^-0.3) X46

X12' = 2.642754 (X11^0.5) (X12^-0.3) X46 -1.642312 (X9^-0.3) (X12^0.5) (X14^-0.3) X48 -4.198429 (X12^0.5)

X13' = 0.004693986 (X10^0.5) X46 X47 +1.642312 (X9^-0.3) (X12^0.5) (X14^-0.3) X48 -30.85202 (X13^0.5)

X14' = 0.6772013 (X9^0.5) (X12^-0.3) (X14^-0.3) X48 +1.642312 (X9^-0.3) (X12^0.5) (X14^-0.3) X48 -168.9835 (X14^0.5)

X15' = 0.0009917856 (X7^0.5) X37 -1.173497 (X15^0.5)

X16' = 0.03710922 X7 (X30^0.5) +0.0009917856 (X7^0.5) X36 +0.0009917856 (X7^0.5) X37 +0.00044354 (X7^0.5) (X28^0.5) X38 -14.10638 (X16^0.5)

X17' = 12.69575 (X16^0.5) -0.1269575 (X17^0.5) X52

X18' = 6.188713 (X5^0.5) -2.767677 (X5^0.5) (X18^0.5)

X19' = 2.767677 (X5^0.5) (X18^0.5) -0.005569842 (X4^0.5) (X19^0.5) X36 -0.006188713 (X19^0.5) X43

X20' = 0.005569842 (X4^0.5) (X19^0.5) X36 +0.1269575 (X17^0.5) X52 -0.02352434 (X4^0.5) (X20^0.5) X36

X21' = 0.02352434 (X4^0.5) (X20^0.5) X36 -23.52434 (X21^0.5)

X22' = 0.006188713 (X19^0.5) X43 -0.6188713 (X22^0.5)

X23' = 23.52434 (X21^0.5) +0.6188713 (X22^0.5) -17.07183 (X23^0.5)

X24' = 0.001785214 (X7^0.5) X40 X41 -15.07592 (X24^0.5) (X25^-0.3) X39

X25' = 15.07592 (X24^0.5) (X25^-0.3) X39 -0.3578389 X25 (X30^0.5) -0.02677821 (X25^0.5) X36 -0.004463035 (X25^0.5) X45 -0.3155842 (X25^0.5) (X31^0.5) -0.6311685 (X25^0.5) (X32^0.5)

X26' = 0.3578389 X25 (X30^0.5) +0.02677821 (X25^0.5) X36 +0.004463035 (X25^0.5) X45 +0.3155842 (X25^0.5) (X31^0.5) +0.6311685 (X25^0.5) (X32^0.5) -149.3617 (X26^0.5)

X27' = 0.6956788 X4 (X30^0.5) +0.07421844 X7 (X30^0.5) +0.7156778 X25 (X30^0.5) -3.065113 (X27^0.5) X45 -38.13551 X27 -0.171345 X27 X28

X28' = 0.001785214 (X7^0.5) X40 X41 +1.532556 (X27^0.5) X45 +33.41228 X27 -1.308324 (X28^0.5) X44 -1.308324 (X28^0.5) X49 -78.01336 X28 -0.171345 X27 X28 -0.00044354 (X7^0.5) (X28^0.5) X38

X29' = 0.004693986 (X10^0.5) X46 X47 +0.001049607 (X9^0.5) X47 X51 -296.8737 (X29^0.5)

X30' = 39.00668 X28 +0.171345 X27 X28 -140.9384 (X30^0.5)

X31' = 4.723232 X27 -0.01686869 X25 X31

X32' = 23.61616 +0.01686869 X25 X31 -0.03373737 X25 X32

X33' = 1.308324 (X28^0.5) X49 -0.2925501 (X33^0.5) X50

X34' = 16.70614 X27 +39.00668 X28 -27.85641 (X34^0.5)

X1 = 5000 //Tyrosine

X2 = 10 //Dihydrobiopterin

X3 = 30 //Tyramine

X4 = 100 //L-DOPA produced from tyrosine

X5 = 5 //Dopaquinone

X6 = 20 //Pyrrolo-quinoline quinone

X7 = 700 //Intracellular dopamine

X8 = 53900 //Dopamine packed in vesicles

X9 = 400 //Extracellular dopamine

X10 = 20 //3-Methoxytyramine

X11 = 10 //Extracellular DOPAL

X12 = 100 //Extracellular DOPAC

X13 = 150 //HVA

X14 = 5 //S-Adenosyl-L-homocysteine

X15 = 5 //Prostaglandin H2

X16 = 10 //Dopamine quinone

X17 = 10 //Dopamine chrome

X18 = 5 //2-Carboxy-2,3-dihydro-5,6-dihydroxyindole

X19 = 5 //L-Dopachrome

X20 = 5 //5,6-Dihydroxyindole

X21 = 5 //Indole-5,6-quinone

X22 = 5 //5,6-Dihydroxyindole-2-carboxylate

X23 = 10 //Melanin

X24 = 5 //Intracellular DOPAL

X25 = 700 //Intracellular DOPAC

X26 = 10 //DOPAC quinone

X27 = 5 //Superoxide

X28 = 5 //Intracellular hydrogen peroxide

X29 = 2 //Extracellular hydrogen peroxide

X30 = 2 //hydroxyl radical

X31 = 2 //Peroxynitrite

X32 = 2 //Nitrogen dioxide

X33 = 100 //Oxidized glutathione

X34 = 100 //Dehydroascorbate

X35 = 100 //Tyrosine hydroxylase

X36 = 100 //Tyrosinase

X37 = 100 //Prostaglandin G/H synthase

X38 = 100 //XO xanthine oxidase

X39 = 100 //ALDH aldehyde dehydrogenase

X40 = 100 //Monoamine oxidase

X41 = 100 //Semicarbazide-sensitive amine oxidase

X42 = 100 //DOPA decarboxylase

X43 = 100 //Dopachrome isomerase

X44 = 100 //Catalase

X45 = 100 //Superoxide dismutase

X46 = 100 //Extracellular aldehyde dehydrogenase

X47 = 100 //Extracellular monoamine oxidase

X48 = 100 //Catechol O-methyltransferase

X49 = 100 //Glutathione peroxidase

X50 = 100 //Glutathione reductase

X51 = 100 //Extracellular semicarbazide-sensitive amine oxidas

X52 = 100 //Migration inhibitory factor

t0 = 0

tf = 5000

hr = 1

***References***
