# Supplementary material for: Computational Systems Analysis of Dopamine Metabolism
Source: PLoS One. 2008 Jun 18;3(6):e2444. doi: 10.1371/journal.pone.0002444 (PMC2435046; doi:10.1371/journal.pone.0002444)
Supplement: Table S1 — Metabolite concentrations and fluxes at steady state (relative units) (0.06 MB DOC) [file pone.0002444.s002.doc]

**Table S1. Metabolite concentrations and fluxes at steady state** (relative units)

| **Variable** | Metabolite | **Abbrevation** | **Concentration** | **Flux** |
| --- | --- | --- | --- | --- |
| *X*_1_ | Tyrosine | Tyr | 5000 | 1000 |
| ***X*_11_** | Dihydrobiopterin | BH2 | 10 | 950 |
| ***X*_12_** | Tyramine |  | 30 | 10 |
| ***X*_2_** | L-DOPA | DOPA | 100 | 983.838 |
| ***X*_14_** | Dopaquinone | DOPA-Q | 5 | 69.192 |
| ***X*_35_** | Pyrrolo-quinoline quinine | PQQ | 20 | 40.823 |
| ***X*_3_** | Dopamine | DA-i | 700 | 2624.020 |
| ***X*_32_** | Dopamine (in vesicles) | DA-v | 53900 | 2099.210 |
| ***X*_33_** | Dopamine (extracellular) | DA-e | 400 | 2099.210 |
| ***X*_16_** | 3-Methoxytyramine | 3-MT | 20 | 209.921 |
| ***X*_17_** | DOPAL (extracellular) | DOPAL-e | 10 | 209.921 |
| ***X*_26_** | DOPAC (extracellular) | DOPAC-e | 100 | 209.921 |
| ***X*_27_** | Homovanillate | HVA | 150 | 377.859 |
| ***X*_20_** | S-Adenosyl-L-homocysteine |  | 5 | 377.859 |
| ***X*_42_** | Prostaglandin H2 | PGH2 | 5 | 2.624 |
| ***X*_30_** | Dopamine Quinone | DA-Q | 10 | 44.608 |
| ***X*_34_** | Dopamine Chrome |  | 10 | 40.148 |
| ***X*_36_** | 2-Carboxy-2,3-dihydro-5,6-dihyroxyindole |  | 5 | 13.838 |
| ***X*_40_** | L-Dopachrome |  | 5 | 13.838 |
| ***X*_37_** | 5,6-Dihydroxyindole |  | 5 | 52.602 |
| ***X*_38_** | Indole-5,6-quinone |  | 5 | 52.602 |
| ***X*_39_** | 5,6-Dihydroxyindole-2-carboxylate | DHICA | 5 | 1.384 |
| ***X*_4_** | Melanin |  | 10 | 53.986 |
| ***X*_23_** | DOPAL |  | 5 | 472.323 |
| ***X*_24_** | DOPAC | DOPAC | 700 | 472.323 |
| ***X*_28_** | DOPAC Quinone | DOPAC-Q | 10 | 472.323 |
| ***X*_70_** | O_2_**^-.^** |  | 5 | 880.341 |
| ***X*_71_** | H_2_O_2_ |  | 5 | 982.075 |
| ***X*_72_** | H_2_O_2_ (extracellular) | H_2_O_2_-e | 2 | 419.843 |
| ***X*_73_** | HO**.** |  | 2 | 199.317 |
| ***X*_76_** | HO**.**---NO_2_**.** |  | 2 | 23.616 |
| ***X*_77_** | **.**NO_2_ |  | 2 | 47.232 |
| ***X*_79_** | Oxidized glutathione | GSSG | 100 | 292.550 |
| ***X*_81_** | Dehydroascorbate |  | 100 | 278.564 |
