# Supplementary material for: Computational Systems Analysis of Dopamine Metabolism
Source: PLoS One. 2008 Jun 18;3(6):e2444. doi: 10.1371/journal.pone.0002444 (PMC2435046; doi:10.1371/journal.pone.0002444)
Supplement: Table S2 — List of independent variables (include environmental factors and enzymes) (0.06 MB DOC) [file pone.0002444.s003.doc]

**Table S2.** List of independent variables (include environmental factors and enzymes)

| **Variable** | **Metabolite** | **Abbreviation** |
| --- | --- | --- |
| *X*_10_ | Tetrahydrobiopterin | BH4 |
| ***X*_13_** | L-Glutamate | Glu |
| ***X*_18_** | S-Adenosyl-L-methionine | SAM |
| ***X*_41_** | Prostaglandin G2 | PGG2 |
| ***X*_74_** | **.**NO | **NO** |
| ***X*_78_** | Glutathione | GSH |
| ***X*_80_** | Ascorbate | ASB |
| ***X*_82_** | N-acetylcysteine |  |
| ***X*_83_** | Fe2+ |  |
| ***X*_84_** | Fe3+ |  |
| ***X*_85_** | NADH |  |
| ***X*_86_** | NAD+ |  |
| ***X*_87_** | NADPH |  |
| ***X*_88_** | NADP+ |  |
| ***X*_89_** | VMAT2 |  |
| ***X*_90_** | DAT |  |
| ***X*_91_** | ATP |  |
| ***X*_50_** | tyrosine hydroxylase | TH |
| ***X*_52_** | tyrosinase | TYR |
| ***X*_53_** | prostaglandin G/H synthase |  |
| ***X*_54_** | xanthine oxidase | XO |
| ***X*_55_** | aldehyde dehydrogenase | ALDH |
| ***X*_56_** | monoamine oxidase | MAO |
| ***X*_57_** | semicarbazide-sensitive amine oxidase | SSAO |
| ***X*_59_** | DOPA decarboxylase | AADC |
| ***X*_60_** | dopachrome isomerase | DCT |
| ***X*_61_** | catalase | CAT |
| ***X*_62_** | superoxide dismutase | SOD |
| ***X*_63_** | aldehyde dehydrogenase (extracellular) | ALDH-e |
| ***X*_64_** | monoamine oxidase (extracellular) | MAO-e |
| ***X*_65_** | catechol O-methyltransferase | COMT |
| ***X*_66_** | glutathione peroxidase | GPx |
| ***X*_67_** | glutathione reductase |  |
| ***X*_68_** | semicarbazide-sensitive amine oxidase (extracellular) | SSAO-e |
| ***X*_69_** | migration inhibitory factor | MIF |
