# Supplementary material for: Computational Systems Analysis of Dopamine Metabolism
Source: PLoS One. 2008 Jun 18;3(6):e2444. doi: 10.1371/journal.pone.0002444 (PMC2435046; doi:10.1371/journal.pone.0002444)
Supplement: Table S3 — Among dozens of independent variables, only DOPA decarboxylase (AACD) significantly affects DOPA concentration, with a log gain of −1.63%, which means that DOPA is predicted to exhibit a 1.63% decrease in response to 1% increase in AADC activity. Dopamine, no matter where it is located (intracellular, in vesicles, or extracellular) is negatively affected by enhancement of MAO or semicarbazide-sensitive amine oxidase (SSAO), which are enzymes catalyzing the degradation of dopamine. Except for MAO and SSAO, the vesicular monoamine transporter (VMAT2) also influences the concentration of DA-v and DA-e, but with positive log gains, while DAT has a negative effect on DA-e. Only Fe2+ has a significant influence on DOPAC, with a log gain of −0.74%. Increases in many independent variables, such as S-adenosyl-L-methionine (SAM), DAT, MAO, SSAO, and COMT are predicted to affect DOPAC-e negatively, while VMAT2, extracellular monoamine oxidase (MAO-e), and extracellular semicarbazide-sensitive amine oxidase (SSAO-e), DOPAC-e have positive log gains. HVA is mainly affected by SAM and COMT. Melanin, the source of pigment in dopaminergic neurons, is affected by changes in the concentrations of SAM, Fe2+, VMAT2, DAT, MAO, SSAO, or COMT. # Gain values are given in percent change due to a 1% percent change in an independent variable. * Gain s with absolute values less than 0.5 are discarded. (0.05 MB RTF) [file pone.0002444.s004.doc]

**Table S3. Log gains of primary metabolites in response to alterations in independent variables^#*^**

|  | **DOPA** | **Dopamine** | **DA-v** | **DA-e** | **DOPAC** | **DOPAC-e** | **HVA** | **Melanin** |
| --- | --- | --- | --- | --- | --- | --- | --- | --- |
| SAM |  |  |  |  |  | -0.95 | 0.51 | -0.65 |
| **Fe2+** |  |  |  |  | -0.74 |  |  | 0.96 |
| **VMAT2** |  |  | 0.80 | 0.94 |  | 1.54 |  | -0.72 |
| **DAT** |  |  |  | -0.75 |  | -1.21 |  | 0.58 |
| **MAO** | -0.65 | -1.20 | -1.20 | -1.42 |  | -2.29 |  | -1.16 |
| **SSAO** | -0.65 | -1.20 | -1.20 | -1.42 |  | -2.29 |  | -1.16 |
| **AADC** | -1.63 |  |  |  |  |  |  |  |
| **CAT** |  |  |  |  |  |  |  | -0.60 |
| **MAO-e** |  |  |  |  |  | 1.76 |  |  |
| **COMT** |  |  |  | -0.55 |  | -1.90 | 1.02 | -1.30 |
| **GPx** |  |  |  |  |  |  |  | -0.60 |
| **SSAO-e** |  |  |  |  |  | 1.76 |  |  |

**^#^** Gain values are given in percent change due to a 1% percent change in an independent variable

**^*^** Gains with absolute values less than 0.5 are discarded

Among dozens of independent variables, only DOPA decarboxylase (AACD) significantly affects DOPA concentration, with a log gain of -1.63%, which means that DOPA is predicted to exhibit a 1.63% decrease in response to 1% increase in AADC activity. Dopamine, no matter where it is located (intracellular, in vesicles, or extracellular) is negatively affected by enhancement of MAO or semicarbazide-sensitive amine oxidase (SSAO), which are enzymes catalyzing the degradation of dopamine. Except for MAO and SSAO, the vesicular monoamine transporter (VMAT2) also influences the concentration of DA-v and DA-e, but with positive log gains, while DAT has a negative effect on DA-e. Only Fe^2+^ has a significant influence on DOPAC, with a log gain of -0.74%. Increases in many independent variables, such as S-adenosyl-L-methionine (SAM), DAT, MAO, SSAO, and COMT are predicted to affect DOPAC-e negatively, while VMAT2, extracellular monoamine oxidase (MAO-e), and extracellular semicarbazide-sensitive amine oxidase (SSAO-e), DOPAC-e have positive log gains. HVA is mainly affected by SAM and COMT. Melanin, the source of pigment in dopaminergic neurons, is affected by changes in the concentrations of SAM, Fe^2+^, VMAT2, DAT, MAO, SSAO, or COMT.
