# Supplementary material for: Computational Systems Analysis of Dopamine Metabolism
Source: PLoS One. 2008 Jun 18;3(6):e2444. doi: 10.1371/journal.pone.0002444 (PMC2435046; doi:10.1371/journal.pone.0002444)
Supplement: Table S4 — Log gains of ROS and RNS in response to alterations in independent variables#*. Only some ROS and RNS show significant log gains with respect to the up-regulation of independent variables. For instance, the gain of O2 −. indicates a 1.29% relative decrease upon 1% elevation of SOD. H2O2 and H2O2-e could not be effectively changed by alterations in any of the independent variables. HO. increased 1.46%, 1.13%, and 1.13% in response to 1% up-regulation of Fe2+, MAO, and SSAO, respectively. Increasing .NO or Fe2+ could promote formation of HO.—NO2 . and .NO2, while increases in ASB, CAT, SOD, COMT, or GPx alleviate HO.—NO2 .. # Gain values are given in percent change due to a 1% percent change in an independent variable. * Gains with absolute values less than 0.5 are discarded. (0.04 MB DOC) [file pone.0002444.s005.doc]

**Table S4. Log gains of ROS and RNS in response to alterations in independent variables^#*^**

|  | **O_2_^-.^** | **H_2_O_2_** | **H_2_O_2_-e** | **HO.** | **HO.---NO_2_.** | **.NO_2_** |
| --- | --- | --- | --- | --- | --- | --- |
| **.NO** |  |  |  |  | 0.86 | 0.93 |
| **ASB** |  |  |  |  | -0.55 |  |
| **Fe^2+^** |  |  |  | 1.46 | 0.91 | 0.83 |
| **MAO** | 0.57 | 0.55 | -0.61 | 1.13 | 0.62 |  |
| **SSAO** | 0.57 | 0.55 | -0.61 | 1.13 | 0.62 |  |
| **CAT** |  |  |  | -0.88 | -0.55 |  |
| **SOD** | -1.29 |  |  |  | -1.44 | -0.80 |
| **COMT** | -0.51 |  | 0.73 | -0.77 | -0.65 |  |
| **GPx** |  |  |  | -0.88 | -0.55 |  |

**^#^** Gain values are given in percent change due to a 1% percent change in an independent variable

**^*^** Gains with absolute values less than 0.5 are discarded

Only some ROS and RNS show significant log gains with respect to the up-regulation of independent variables. For instance, the gain of O_2_^-^. indicates a 1.29% relative decrease upon 1% elevation of SOD. H_2_O_2_ and H_2_O_2_-e could not be effectively changed by alterations in any of the independent variables. HO. increased 1.46%, 1.13%, and 1.13% in response to 1% up-regulation of Fe^2+^, MAO, and SSAO, respectively. Increasing .NO or Fe^2+^ could promote formation of HO.---NO_2_. and .NO_2_, while increases in ASB, CAT, SOD, COMT, or GPx alleviate HO.---NO_2_..
