# Supplementary material for: Computational Systems Analysis of Dopamine Metabolism
Source: PLoS One. 2008 Jun 18;3(6):e2444. doi: 10.1371/journal.pone.0002444 (PMC2435046; doi:10.1371/journal.pone.0002444)
Supplement: Table S5 — Log gains of toxic species with respect to alterations in independent variables#*. The most effective way of decreasing toxic DOPA-Q is increasing the activity of AADC; lowering Fe2+ has a similar but lesser effect. 3-MT could be alleviated by elevation of extracellular aldehyde dehydrogenase (ALDH-e) or MAO-e, or reduction of SAM or COMT. DOPAL is mainly affected by Fe2+, NAD+, NADH, NADP+, NADPH, and ALDH. Elevation of DAT, MAO, or SSAO has the most significant negative effect on the concentration of DOPAL-e, while increases in VMAT2, MAO-e, or SSAO-e could promote generation of DOPAL-e. None of these primary metabolites could significantly reduce the concentration of DOPAC-Q. To lessen content of DA-Q, Fe2+ should be decreased or VMAT2, SAM, MAO, SSAO, or COMT increased. However, all effects are only moderate. # Gain values are given in percent change due to a 1% percent change in an independent variable. * Gains with absolute values less than 0.5 are discarded. (0.05 MB DOC) [file pone.0002444.s006.doc]

**Table S5. Log gains of toxic species with respect to alterations in independent variables^#*^**

|  | **DOPA-Q** | **3-MT** | **DOPAL** | **DOPAL-e** | **DOPAC-Q** | **DA-Q** |
| --- | --- | --- | --- | --- | --- | --- |
| Glu | -0.70 |  |  |  |  |  |
| **SAM** | -0.63 | 1.00 |  | -0.84 |  | -0.66 |
| **Fe2+** | 1.04 |  | -0.59 |  |  | 0.93 |
| **NADH** |  |  | 0.60 | 0.60 |  |  |
| **NAD+** |  |  | -0.99 | -0.99 |  |  |
| **NADPH** |  |  | 0.60 | 0.60 |  |  |
| **NADP+** |  |  | -0.99 | -0.99 |  |  |
| **VMAT2** | -0.70 |  |  | 1.87 |  | -0.73 |
| **DAT** | 0.56 |  |  | -1.48 |  | 0.58 |
| **ALDH** |  |  | -1.97 |  |  |  |
| **MAO** | -0.97 |  | 0.76 | -2.78 | 0.79 | -1.23 |
| **SSAO** | -0.97 |  | 0.76 | -2.78 | 0.79 | -1.23 |
| **AADC** | -3.37 |  |  | 0.56 |  | 0.53 |
| **CAT** | -0.63 |  |  |  |  | -0.59 |
| **ALDH-e** |  | -1.97 |  | -1.97 |  |  |
| **MAO-e** |  | -3.23 |  | 2.89 |  |  |
| **COMT** | -1.26 | 2.00 |  | -1.68 |  | -1.31 |
| **GPx** | -0.63 |  |  |  |  | -0.59 |
| **SSAO-e** |  | -1.29 |  | 2.89 |  |  |

**^#^** Gain values are given in percent change due to a 1% percent change in an independent variable

**^*^** Gains with absolute values less than 0.5 are discarded

The most effective way of decreasing toxic DOPA-Q is increasing the activity of AADC; lowering Fe^2+^ has a similar but lesser effect. 3-MT could be alleviated by elevation of extracellular aldehyde dehydrogenase (ALDH-e) or MAO-e, or reduction of SAM or COMT. DOPAL is mainly affected by Fe^2+^, NAD+, NADH, NADP+, NADPH, and ALDH. Elevation of DAT, MAO, or SSAO has the most significant negative effect on the concentration of DOPAL-e, while increases in VMAT2, MAO-e, or SSAO-e could promote generation of DOPAL-e. None of these primary metabolites could significantly reduce the concentration of DOPAC-Q. To lessen content of DA-Q, Fe^2+^ should be decreased or VMAT2, SAM, MAO, SSAO, or COMT increased. However, all effects are only moderate.
