# Supplementary material for: Computational Systems Analysis of Dopamine Metabolism
Source: PLoS One. 2008 Jun 18;3(6):e2444. doi: 10.1371/journal.pone.0002444 (PMC2435046; doi:10.1371/journal.pone.0002444)
Supplement: Table S6 — Sensitivity of primary metabolites in response to alterations in kinetic orders#*. DOPA shows negative gains with respect to kinetic orders for its degradation processes, except for the inhibition from dopamine. The sensitivity of DOPA in response to alteration of dopamine inhibition is 3.24%, which means 1% up-regulation of dopamine inhibition is predicted to cause a 3.24% relative increase in DOPA. Dopamine and DA-v have very similar sensitivity profiles for kinetic orders, with positive values associated with DOPA degradation and negative values associated with degradative processes of dopamine and DA-e (especially for fluxes from dopamine to DOPAL and from DA-e to 3-MT). DA-e is the actual transmitter of nerve signals for movement control. Its only noteworthy sensitivities (with respect to DA-e self-degradation and dopamine degradation toward DOPAL) are negative. Hence, measures to decrease the effects of dopamine, MAO, or SSAO on the reaction between dopamine and DOPAL could potentially constitute efficacious interventions to increase DA-e concentrations. DOPAC is mainly affected by kinetic orders for H2O2 degradation. Sensitivities of DOPAC-e are negatively influenced by dopamine degradation and positively by increases in the reaction between DA-e and DOPAL-e. HVA and melanin show several significant sensitivity values, mostly associated with kinetic orders related to the degradation of DOPA, dopamine, and DA-e. # Sensitivity values are given in percent change due to a 1% percent change in a parameter * Sensitivities with absolute values less than 0.5 are discarded. (0.08 MB DOC) [file pone.0002444.s007.doc]

**Table S6. Sensitivity of primary metabolites in response to alterations in kinetic orders^#*^**

|  | **DOPA** | **Dopamine** | **DA-v** | DA-e | **DOPAC** | **DOPAC-e** | **HVA** | **Melanin** |
| --- | --- | --- | --- | --- | --- | --- | --- | --- |
| **f2_2_01** | -3.67 | 0.54 | 0.54 | 0.64 |  | 1.03 |  | -1.07 |
| **f2_59** | -7.28 | 1.06 | 1.06 | 1.26 |  | 2.06 |  | -2.12 |
| **f2_3** | 3.24 |  |  | -0.56 |  | -0.91 |  | 0.96 |
| **f14_13** |  |  |  |  |  |  |  | -1.57 |
| **f3_3_01** | -2.10 | -3.87 | -3.87 | -4.59 |  | -7.34 | -1.36 | -3.80 |
| **f3_56** | -2.97 | -5.45 | -5.45 | -6.46 |  | -10.26 | -1.92 | -5.36 |
| **f3_57** | -2.97 | -5.45 | -5.45 | -6.46 |  | -10.26 | -1.92 | -5.36 |
| **f33_33_01** |  | -0.78 | -0.78 | -1.17 |  | -0.87 | 1.83 | -2.77 |
| **f33_18** |  |  |  |  |  |  |  | -0.75 |
| **f33_65** |  | -1.21 | -1.21 | -1.81 |  | -1.35 | 2.84 | -4.27 |
| **f33_26** |  |  |  | 0.54 |  |  | -0.83 | 1.29 |
| **f33_33_02** |  |  |  | -0.60 |  | 5.38 |  | -1.42 |
| **f33_64** |  | -0.63 | -0.63 | -0.94 |  | 8.37 |  | -2.22 |
| **f33_68** |  | -0.63 | -0.63 | -0.94 |  | 8.37 |  | -2.22 |
| **f26_26_01** |  |  |  |  |  | -3.68 | 0.95 | -0.86 |
| **f26_18** |  |  |  |  |  | -1.31 |  |  |
| **f26_65** |  |  |  | -0.73 |  | -7.30 | 1.92 | -1.75 |
| **f26_20** |  |  |  |  |  | 0.79 |  |  |
| **f26_33** |  |  |  |  |  | 2.97 | -0.74 | 0.68 |
| **f26_26_02** |  |  |  |  |  | -1.23 |  |  |
| **f27_27** |  |  |  |  |  |  | -4.84 |  |
| **f20_20** |  |  |  |  |  | -0.92 |  | -0.63 |
| **f4_4** |  |  |  |  |  |  |  | -2.25 |
| **f24_24_02** |  |  |  |  |  |  |  | -0.54 |
| **f24_52** |  |  |  |  |  |  |  | -0.77 |
| **f70_62** |  |  |  |  | 0.77 |  |  | -0.79 |
| **f71_61** |  |  |  |  | 2.15 | 0.80 |  | -2.81 |
| **f71_78** | 0.66 | 0.60 | 0.60 | 0.71 | 3.11 | 1.15 |  | -4.01 |
| **f71_66** |  |  |  |  | 2.15 | 0.80 |  | -2.81 |

**^#^** Sensitivity values are given in percent change due to a 1% percent change in a parameter

**^*^** Sensitivities with absolute values less than 0.5 are discarded

DOPA shows negative gains with respect to kinetic orders for its degradation processes, except for the inhibition from dopamine. The sensitivity of DOPA in response to alteration of dopamine inhibition is 3.24%, which means 1% up-regulation of dopamine inhibition is predicted to cause a 3.24% relative increase in DOPA. Dopamine and DA-v have very similar sensitivity profiles for kinetic orders, with positive values associated with DOPA degradation and negative values associated with degradative processes of dopamine and DA-e (especially for fluxes from dopamine to DOPAL and from DA-e to 3-MT). DA-e is the actual transmitter of nerve signals for movement control. Its only noteworthy sensitivities (with respect to DA-e self-degradation and dopamine degradation toward DOPAL) are negative. Hence, measures to decrease the effects of dopamine, MAO, or SSAO on the reaction between dopamine and DOPAL could potentially constitute efficacious interventions to increase DA-e concentrations. DOPAC is mainly affected by kinetic orders for H_2_O_2_ degradation. Sensitivities of DOPAC-e are negatively influenced by dopamine degradation and positively by increases in the reaction between DA-e and DOPAL-e. HVA and melanin show several significant sensitivity values, mostly associated with kinetic orders related to the degradation of DOPA, dopamine, and DA-e.
