# Supplementary material for: Computational Systems Analysis of Dopamine Metabolism
Source: PLoS One. 2008 Jun 18;3(6):e2444. doi: 10.1371/journal.pone.0002444 (PMC2435046; doi:10.1371/journal.pone.0002444)
Supplement: Table S7 — Sensitivity of primary metabolites in response to alterations in rate constants#*. As with kinetic orders, most of the sensitivities with respect to rate constants are negligible in magnitude. Of note is rate constant γ1−0, which represents the exogenous input flux into the dopamine metabolic system. As one might expect, enhancements in this flux yield increases in the concentrations of most of the primary metabolites especially that of melanin, with the exception of DOPAC, which slightly decreases. Almost all other rate constant sensitivities are of magnitude 1 or smaller. DOPA is negatively affected by rate constants for degradation of DOPA and dopamine. Dopamine, DA-v, and DA-e have negative sensitivities with respect to the rate constant for the reaction between dopamine and DOPAL. Increasing the transport of dopamine into vesicles could increase the concentrations of DA-v and DA-e, while enhancing degradation of DA-v and DA-e is expected to lead to decreases in their concentrations, respectively. DOPAC, DOPAC-e and HVA are mainly affected by rate constants related to their synthesis and degradation. DOPAC-e also has significant sensitivities with respect to rate constants for dopamine reactions. Many rate constants influence melanin to some extent but much less than γ1−0. # Sensitivity values are given in percent change due to a 1% percent change in a parameter. * Sensitivities with absolute values less than 0.5 are discarded. (0.06 MB DOC) [file pone.0002444.s008.doc]

**Table S7. Sensitivity of primary metabolites in response to alterations in rate constants^#*^**

|  | **DOPA** | **Dopamine** | **DA-v** | **DA-e** | **DOPAC** | **DOPAC-e** | **HVA** | **Melanin** |
| --- | --- | --- | --- | --- | --- | --- | --- | --- |
| *γ***1_0** | 2.34 | 1.62 | 1.62 | 1.92 | -0.82 | 3.13 | 0.56 | 7.60 |
| *γ***1_01** | -1.61 |  |  |  |  |  |  |  |
| *γ***1_02** |  |  |  | -0.75 |  | -1.21 |  | 0.58 |
| *γ***1_03** |  |  | 0.80 | 0.94 |  | 1.54 |  | -0.72 |
| *γ***1_04** | -0.65 | -1.20 | -1.20 | -1.42 |  | -2.29 |  | -1.16 |
| *γ***11** |  |  |  |  |  |  |  | 1.08 |
| *γ***12** |  |  | -0.99 |  |  |  |  |  |
| *γ***22_01** |  |  |  |  |  |  | 0.60 | -0.92 |
| *γ***22_02** |  |  |  |  |  | 1.76 |  |  |
| *γ***2_03** |  |  |  |  |  | -1.62 |  |  |
| *γ***2_01** |  |  |  |  |  | -0.53 |  |  |
| *γ***2_02** |  |  |  |  |  |  | -1.97 |  |
| *γ***14_01** |  |  |  |  |  | -1.14 | 0.61 | -0.78 |
| *γ***14_02** |  |  |  |  |  |  |  | -1.97 |
| *γ***14_03** |  |  |  |  | -0.96 |  |  |  |
| *γ***14_04** |  |  |  |  |  |  |  | -0.60 |
| *γ***35** |  |  |  |  |  |  |  | -0.60 |
| *γ***3_03** |  |  |  |  | -0.74 |  |  | 0.96 |
| *γ***3_00** |  |  |  |  | 1.07 |  |  | -1.38 |

**^#^** Sensitivity values are given in percent change due to a 1% percent change in a parameter

**^*^** Sensitivities with absolute values less than 0.5 are discarded

As with kinetic orders, most of the sensitivities with respect to rate constants are negligible in magnitude. Of note is rate constant *γ*_1-0_, which represents the exogenous input flux into the dopamine metabolic system. As one might expect, enhancements in this flux yield increases in the concentrations of most of the primary metabolites especially that of melanin, with the exception of DOPAC, which slightly decreases. Almost all other rate constant sensitivities are of magnitude 1 or smaller. DOPA is negatively affected by rate constants for degradation of DOPA and dopamine. Dopamine, DA-v, and DA-e have negative sensitivities with respect to the rate constant for the reaction between dopamine and DOPAL. Increasing the transport of dopamine into vesicles could increase the concentrations of DA-v and DA-e, while enhancing degradation of DA-v and DA-e is expected to lead to decreases in their concentrations, respectively. DOPAC, DOPAC-e and HVA are mainly affected by rate constants related to their synthesis and degradation. DOPAC-e also has significant sensitivities with respect to rate constants for dopamine reactions. Many rate constants influence melanin to some extent but much less than *γ*_1-0_.
