# Supplementary material for: Computational Systems Analysis of Dopamine Metabolism
Source: PLoS One. 2008 Jun 18;3(6):e2444. doi: 10.1371/journal.pone.0002444 (PMC2435046; doi:10.1371/journal.pone.0002444)
Supplement: Table S8 — Sensitivities of ROS and RNS in response to alterations of kinetic orders#*. Enhancing the degradation of dopamine increases all ROS and RNS except H2O2-e, while elevation of kinetic orders for effluxes out of DA-e reduce the concentrations of most ROS and RNS. O2 -., H2O2, and H2O2-e decrease upon up-regulation of their relevant degradation processes. Increasing kinetic orders for degradation of DOPAC, DOPAC-e, O2 -., H2O2, or HO. alleviates HO. concentration. HO. —NO2 . and . NO2 could be decreased by enhancing the degradation of O2 -. or H2O2. Enzymes and antioxidants within the cell defense system, such as CAT, SOD, GPx, and GSH, exhibit kinetic order sensitivities that show moderate capability of scavenging these reactive species. (0.05 MB DOC) [file pone.0002444.s009.doc]

**Table S8. Sensitivities of ROS and RNS in response to alterations of kinetic orders^#*^**

|  | **O_2_^-.^** | **H_2_O_2_** | **H_2_O_2_-e** | **HO.** | **HO.---NO_2_.** | **.NO_2_** |
| --- | --- | --- | --- | --- | --- | --- |
| **f2_59** |  |  | 0.54 |  | -0.66 | -0.54 |
| **f3_3_01** | 1.85 | 1.79 | -1.97 | 3.70 | 2.01 | 1.08 |
| **f3_56** | 2.62 | 2.53 | -2.77 | 5.25 | 2.84 | 1.53 |
| **f3_57** | 2.62 | 2.53 | -2.77 | 5.25 | 2.84 | 1.53 |
| **f33_33_01** | -1.10 | -0.80 | 1.56 | -1.64 | -1.4 | -0.85 |
| **f33_65** | -1.70 | -1.24 | 2.42 | -2.53 | -2.16 | -1.31 |
| **f33_26** |  |  | -0.71 | 0.75 | 0.64 |  |
| **f33_33_02** | -0.56 |  | 0.79 | -0.84 | -0.71 |  |
| **f33_64** | -0.88 | -0.64 | 1.25 | -1.31 | -1.12 | -0.68 |
| **f33_68** | -0.88 | -0.64 | 1.25 | -1.31 | -1.12 | -0.68 |
| **f26_26_01** |  |  |  | -0.51 |  |  |
| **f26_65** | -0.69 |  | 0.98 | -1.03 | -0.88 | -0.53 |
| **f24_24_02** | -0.84 |  |  | -0.8 | -0.67 |  |
| **f24_52** | -1.19 | -0.54 |  | -1.13 | -0.95 |  |
| **f70_70_01** | -1.00 |  |  |  | -1.13 | -0.63 |
| **f70_62** | -5.69 |  |  | -1.17 | -6.41 | -3.59 |
| **f71_71_01** |  |  |  | -0.71 |  |  |
| **f71_61** |  | -2.05 |  | -4.08 | -2.54 | -2.32 |
| **f71_71_02** |  |  |  | -0.71 |  |  |
| **f71_78** | -0.64 | -2.94 |  | -5.82 | -3.64 | -3.32 |
| **f71_66** |  | -2.05 |  | -4.08 | -2.54 | -2.32 |
| **f72_72** |  |  | -0.68 |  |  |  |
| **f73_73** |  |  |  | -0.72 |  |  |

**^#^** Sensitivity values are given in percent change due to a 1% percent change in a parameter

**^*^** Sensitivities with absolute values less than 0.5 are discarded

Enhancing the degradation of dopamine increases all ROS and RNS except H_2_O_2_-e, while elevation of kinetic orders for effluxes out of DA-e reduce the concentrations of most ROS and RNS. O_2_**^-.^**, H_2_O_2_, and H_2_O_2_-e decrease upon up-regulation of their relevant degradation processes. Increasing kinetic orders for degradation of DOPAC, DOPAC-e, O_2_**^-.^**, H_2_O_2_, or HO**.** alleviates HO**.** concentration. HO**.**---NO_2_**.** and **.**NO_2_ could be decreased by enhancing the degradation of O_2_**^-.^** or H_2_O_2_. Enzymes and antioxidants within the cell defense system, such as CAT, SOD, GPx, and GSH, exhibit kinetic order sensitivities that show moderate capability of scavenging these reactive species.
