# Supplementary material for: Computational Systems Analysis of Dopamine Metabolism
Source: PLoS One. 2008 Jun 18;3(6):e2444. doi: 10.1371/journal.pone.0002444 (PMC2435046; doi:10.1371/journal.pone.0002444)
Supplement: Table S9 — Increasing rate constant γ1−0 would raise the concentrations of all ROS and RNS, especially those of HO. and HO.—NO2 .; the rate constants for degradation of dopamine to DOPAL have similar but lesser effects. Most of ROS and RNS could be significantly alleviated by enhancing their own degradation. HO. could also be reduced through enhancement of H2O2 degradation. Increasing the rate constant for DOPAC autoxidation would elevate HO.—NO2 . and .NO2, while rate constants for O2 -. and HO. degradation have the opposite effect. (0.04 MB DOC) [file pone.0002444.s010.doc]

**Table S9. Sensitivities of ROS and RNS in response to alterations of rate constants^#*^**

|  | **O_2_^-.^** | **H_2_O_2_** | **H_2_O_2_-e** | **HO.** | **HO.---NO_2_.** | **.NO_2_** |
| --- | --- | --- | --- | --- | --- | --- |
| *γ***1_0** | 2.69 | 1.86 | 0.82 | 3.87 | 3.54 | 2.18 |
| *γ***3_01** | 0.57 | 0.55 | -0.61 | 1.13 | 0.62 |  |
| *γ***33_01** |  |  | 0.51 | -0.54 |  |  |
| *γ***24_01** |  |  |  |  | 1.12 | 1.04 |
| *γ***70_04** |  |  |  |  | 0.89 |  |
| *γ***70_01** | -1.24 |  |  |  | -1.41 | -0.79 |
| *γ***71_01** |  |  |  | -0.88 | -0.55 |  |
| *γ***71_02** |  |  |  | -0.88 | -0.55 |  |
| *γ***71_03** |  |  |  | 1.46 | 0.91 | 0.83 |
| *γ***71_04** |  |  |  | -0.59 |  |  |
| *γ***72** |  |  | -1.97 |  |  |  |
| *γ***73** |  |  |  | -2.07 | -1.29 | -1.18 |
| *γ***76** |  |  |  |  | -0.97 |  |
| *γ***77** |  |  |  |  |  | -0.98 |

**^#^** Sensitivity values are given in percent change due to a 1% percent change in a parameter

**^*^** S Sensitivities with absolute values less than 0.5 are discarded

Increasing rate constant *γ*_1-0_ would raise the concentrations of all ROS and RNS, especially those of HO**.** and HO**.**---NO_2_**.**; the rate constants for degradation of dopamine to DOPAL have similar but lesser effects. Most of ROS and RNS could be significantly alleviated by enhancing their own degradation. HO**.** could also be reduced through enhancement of H_2_O_2_ degradation. Increasing the rate constant for DOPAC autoxidation would elevate HO**.**---NO_2_**.** and **.**NO_2_, while rate constants for O_2_**^-.^** and HO**.** degradation have the opposite effect.
