# Supplementary material for: Computational Systems Analysis of Dopamine Metabolism
Source: PLoS One. 2008 Jun 18;3(6):e2444. doi: 10.1371/journal.pone.0002444 (PMC2435046; doi:10.1371/journal.pone.0002444)
Supplement: Table S10 — Sensitivity of toxic species in response to alteration of kinetic orders#*. Quite a few kinetic orders could efficaciously decrease the concentration of DOPA-Q, especially those related to L-DOPA conversion to dopamine and DOPA-Q degradation to Pyrrolo-quinoline quinine (PQQ). 3-MT, DOPAL, and DOPAC-Q have significant sensitivities with respect to kinetic orders related to their degradation. Reduction in kinetic orders for DA-e degradation to 3-MT could also effectively alleviate the concentration of 3-MT. Increasing the effect of dopamine, MAO, or SSAO on dopamine degradation could greatly alleviate the concentration of DOPAL-e; decreasing the kinetic order for the reaction between DA-e and DOPAL-e has a similar effect. Increases in DOPAL-e degradation could somewhat reduce its own concentration. DA-Q shows significant sensitivities mostly with respect to kinetic orders for the conversion of dopamine to DOPAL. Elevation of the action of GSH on H2O2 could indirectly result in great reductions of toxic DA-Q. # Sensitivity values are given in percent change due to a 1% percent change in a parameter. * Sensitivities with absolute values less than 3.0 are discarded. (0.05 MB DOC) [file pone.0002444.s011.doc]

**Table S10. Sensitivity of toxic species in response to alteration of kinetic orders^#*^**

|  | **DOPA-Q** | **3-MT** | **DOPAL** | **DOPAL-e** | **DOPAC-Q** | **DA-Q** |
| --- | --- | --- | --- | --- | --- | --- |
| **f2_2_01** | -7.49 |  |  |  |  |  |
| **f2_59** | -14.60 |  |  |  |  |  |
| **f3_3_01** | -3.15 |  |  | -8.86 |  | -4.02 |
| **f3_56** | -4.45 |  | 3.54 | -12.34 | 3.68 | -5.68 |
| **f3_57** | -4.45 |  | 3.54 | -12.34 | 3.68 | -5.68 |
| **f33_33_01** |  | 4.33 |  |  |  |  |
| **f33_65** | -4.15 | 6.74 |  |  |  | -4.30 |
| **f33_64** |  | -3.84 |  | 8.90 |  |  |
| **f33_68** |  | -5.87 |  | 13.99 |  |  |
| **f16_16** |  | -5.87 |  | 13.99 |  |  |
| **f16_64** |  | -8.80 |  |  |  |  |
| **f17_17** |  | -8.80 |  |  |  |  |
| **f17_88** |  |  |  | -4.50 |  |  |
| **f17_63** |  |  |  | -4.50 |  |  |
| **f17_26** |  |  |  | -8.80 |  |  |
| **f23_86** |  |  |  | -5.15 |  |  |
| **f23_55** |  |  | -4.50 |  |  |  |
| **f23_24** |  |  | -4.50 |  |  |  |
| **f23_85** |  |  | -8.80 |  |  |  |
| **f23_87** |  |  | 4.01 |  |  |  |

**^#^** Sensitivity values are given in percent change due to a 1% percent change in a parameter

**^*^** S Sensitivities with absolute values less than 3.0 are discarded

Quite a few kinetic orders could efficaciously decrease the concentration of DOPA-Q, especially those related to L-DOPA conversion to dopamine and DOPA-Q degradation to Pyrrolo-quinoline quinine (PQQ). 3-MT, DOPAL, and DOPAC-Q have significant sensitivities with respect to kinetic orders related to their degradation. Reduction in kinetic orders for DA-e degradation to 3-MT could also effectively alleviate the concentration of 3-MT. Increasing the effect of dopamine, MAO, or SSAO on dopamine degradation could greatly alleviate the concentration of DOPAL-e; decreasing the kinetic order for the reaction between DA-e and DOPAL-e has a similar effect. Increases in DOPAL-e degradation could somewhat reduce its own concentration. DA-Q shows significant sensitivities mostly with respect to kinetic orders for the conversion of dopamine to DOPAL. Elevation of the action of GSH on H_2_O_2_ could indirectly result in great reductions of toxic DA-Q.
