# Supplementary material for: Computational Systems Analysis of Dopamine Metabolism
Source: PLoS One. 2008 Jun 18;3(6):e2444. doi: 10.1371/journal.pone.0002444 (PMC2435046; doi:10.1371/journal.pone.0002444)
Supplement: Table S11 — Sensitivity of toxic species in response to alteration of rate constants#*. Rate constant γ1−0, which accounts for the exogenous input flux into the dopamine system has a strong positive effect on the concentrations of DOPA-Q, DOPAL-e, and DA-Q. Enhancing the degradation of DOPA could decrease DOPA-Q moderately compared with that from γ1−0. To effectively reduce the concentrations of 3-MT, DOPAL, and DOPAC-Q, their relevant rate constants for degradative processes should be raised. DOPAL-Q could be reduced by increasing the rate constant for conversion of dopamine to DOPAL or slowing down degradation of DA-e to DOPAL-e. The rate constant for DA-Q degradation has a negative effect on the concentration of DA-Q but with smaller magnitude in comparison with γ1−0. # Sensitivity values are given in percent change due to a 1% percent change in a parameter * Sensitivities with absolute values less than 0.5 are discarded. (0.06 MB DOC) [file pone.0002444.s012.doc]

**Table S11. Sensitivity of toxic species in response to alteration of rate constants^#*^**

|  | **DOPA-Q** | **3-MT** | **DOPAL** | **DOPAL-e** | **DOPAC-Q** | **DA-Q** |
| --- | --- | --- | --- | --- | --- | --- |
| *γ***1_0** | 11.23 |  | 1.12 | 3.82 | 1.62 | 6.36 |
| *γ***2_01** | -3.33 |  |  | 0.55 |  | 0.52 |
| *γ***2_02** | 1.34 |  |  |  |  |  |
| *γ***14_01** | -1.40 |  |  |  |  |  |
| *γ***14_02** | -0.81 |  |  |  |  |  |
| *γ***3_03** | 0.56 |  |  | -1.48 |  | 0.58 |
| *γ***3_00** | -0.70 |  |  | 1.87 |  | -0.73 |
| *γ***3_01** | -0.97 |  | 0.76 | -2.78 | 0.79 | -1.23 |
| *γ***3_02** |  |  |  |  |  | 1.44 |
| *γ***33_01** | -0.90 | 1.42 |  | -0.56 |  | -0.93 |
| *γ***33_02** |  | -1.29 |  | 2.89 |  |  |
| *γ***16** |  | -1.97 |  |  |  |  |
| *γ***17** |  |  |  | -1.97 |  |  |
| *γ***26_01** |  | 0.57 |  | -1.13 |  |  |
| *γ***20** | -0.76 | 1.20 |  | -1.01 |  | -0.79 |
| *γ***30_01** |  |  |  |  |  | -1.72 |
| *γ***23** |  |  | -1.97 |  |  |  |
| *γ***24_01** |  |  | -0.59 |  |  |  |
| *γ***28** |  |  |  |  | -1.97 |  |
| *γ***71_01** | -0.63 |  |  |  |  | -0.59 |
| *γ***71_02** | -0.63 |  |  |  |  | -0.59 |
| *γ***71_03** | 1.04 |  | -0.59 |  |  | 0.93 |
| *γ***73** | -1.48 |  | 0.85 |  |  | -1.34 |

**^#^** Sensitivity values are given in percent change due to a 1% percent change in a parameter

**^*^** S Sensitivities with absolute values less than 0.5 are discarded

Rate constant *γ*_1-0_, which accounts for the exogenous input flux into the dopamine system has a strong positive effect on the concentrations of DOPA-Q, DOPAL-e, and DA-Q. Enhancing the degradation of DOPA could decrease DOPA-Q moderately compared with that from *γ*_1-0_. To effectively reduce the concentrations of 3-MT, DOPAL, and DOPAC-Q, their relevant rate constants for degradative processes should be raised. DOPAL-Q could be reduced by increasing the rate constant for conversion of dopamine to DOPAL or slowing down degradation of DA-e to DOPAL-e. The rate constant for DA_Q degradation has a negative effect on the concentration of DA-Q but with smaller magnitude in comparison with *γ*_1-0_.
